# A Review of Heating and Temperature Control in Microfluidic Systems: Techniques and Applications

**DOI:** 10.3390/diagnostics3010033

**Published:** 2013-01-15

**Authors:** Vincent Miralles, Axel Huerre, Florent Malloggi, Marie-Caroline Jullien

**Affiliations:** 1 Gulliver CNRS ESPCI, UMR7083, MMN, 10 rue Vauquelin, 75005 Paris, France; E-Mails: vincent.miralles@espci.fr (V.M.); axel.huerre@espci.fr (A.H.); 2 SIS2M-LIONS CEA CNRS, UMR 3299, CEA Saclay, 91191 Gif-sur-Yvette, France; E-Mail: florent.malloggi@cea.fr

**Keywords:** heating, temperature, microfluidics

## Abstract

This review presents an overview of the different techniques developed over the last decade to regulate the temperature within microfluidic systems. A variety of different approaches has been adopted, from external heating sources to Joule heating, microwaves or the use of lasers to cite just a few examples. The scope of the technical solutions developed to date is impressive and encompasses for instance temperature ramp rates ranging from 0.1 to 2,000 °C/s leading to homogeneous temperatures from −3 °C to 120 °C, and constant gradients from 6 to 40 °C/mm with a fair degree of accuracy. We also examine some recent strategies developed for applications such as digital microfluidics, where integration of a heating source to generate a temperature gradient offers control of a key parameter, without necessarily requiring great accuracy. Conversely, Temperature Gradient Focusing requires high accuracy in order to control both the concentration and separation of charged species. In addition, the Polymerase Chain Reaction requires both accuracy (homogeneous temperature) and integration to carry out demanding heating cycles. The spectrum of applications requiring temperature regulation is growing rapidly with increasingly important implications for the physical, chemical and biotechnological sectors, depending on the relevant heating technique.

## 1. Introduction

The development of lab-on-a-chip requires the integration of multiple functions within a compact platform, which is readily transportable and can deliver rapid data output. One such functionality is the control of temperature, either in terms of profile (homogeneous or gradient) or in terms of the accessible range, and in both cases with the greatest accuracy possible. Indeed, the regulation of temperature is a critical parameter in managing many physical, chemical and biological applications. Prominent examples of applications requiring tight temperature control are Polymerase Chain Reaction (PCR) [1,2,3,4,5,6,7,8,9,10,11,12,13,14,15], Temperature Gradient Focusing for Electrophoresis (TGF) [16,17], digital microfluidics [18,19,20,21,22,23,24,25,26,27,28,29,30,31], mixing [32,33,34], and protein crystallization [35]. The scope of this article is to provide a comprehensive applications-based overview of heating techniques reported in the literature during the last decade. The vast majority of studies, involving heating/cooling technologies exploit external approaches such as the use of macroscopic Peltier or pre-heated liquids [1,2,3,4,5,36,37,38,39,40] flowing through the microsystem. These technologies facilitate both homogeneous temperature regulation within the whole microsystem, and linear temperature profiles often with a high degree of accuracy; however the control is not integrated and may thus limit the potential applications. In addition to the integration of micro-Peltier components, other integrated technologies have been developed using: Joule heating [15,20,21,28,29,30,41,42,43,44,45,46], microwaves [47,48,49,50,51,52,53,54,55,56,57,58,59,60,61,62,63,64], endothermal chemical reactions [65,66], and integrated wires and lasers [67,68,69,70]. 

The review is organized as follows: 

The first sections focus on the different techniques reported to date. These techniques are described along with the corresponding specifications if explicitly stated in the original report (see Table 1). Techniques are classified according to their level of integration:

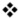
 Section 2 covers external heating methods, *i.e*., by means of commercial heaters with some degree of integration, but entirely external approaches (such as using hot-plates) are not considered here,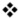 Section 3 using integrated heaters within the microsystem, and,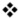 Section 4 heating techniques exploiting electromagnetic radiations*, i.e.*, the liquid is directly heated in the bulk material.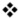 In Section 5, we introduce a range of applications that illustrate the efficacy of different heating techniques. Throughout the paper we refer to these applications, the reader not familiar with the applications is invited to refer to this section. The spectrum of applications is wide-ranging and rapidly growing; and it is not our intention to detail all the applications that may benefit from thermal control but rather to give an overview of the variety of approaches that have been adapted for different applications.

**Table 1:** Summary of the specifications related to the techniques discussed in the review.

Heating method					Level of integration	Range of temperature (°C)	Spatial distribution				Power needed (mW)
Pre-heated liquids	Joule heating		Microwaves	Chemical reactions			Constant T		Gradient T		
	homogeneous T	gradient T					Ramp rate (°C/s)	Accuracy (± °C)	Maximum value (°C/mm)	Response time	
[36]					+	5–45	+4 −3	0.3			
[37]					+	5–45	+4 −3	1			
[38]					+ +	10–80			5.8		
[46]					+ +	10–80			25		<50
[16]					+ +	5–60			13.75	~3 min	
[40]					+ + +	−3–120	+106 −89	0.2			
[2]					+ + +	22–95	+100 −90	0.1			
	[67]				+ +	20–96	+20 −11.5	0.5			1,000
	[41]				+ +	20–130	+0.1	0.2			
	[44]				+ + +	50–100	+20 −10	1			2,200
	[42]				+ +	25–130					1,700
	[15]				+ + +	25–96					
	[13]				+ + +	25–96	+20 −10	0.2			
	[29]				+ + +	25–55			11	1 s	500
	[45]				+ + +	25–96	+20 −20	0.6			300
		[20]			+ + +	25–96			6	~1 min	500
		[21]			+ + +	25–96			40		500
		[43]			+ +	25–75	+20	2			1,000
			[59]		+	25–32					10
			[56]		+	20–70	+7	not stable			400
			[57]		+	20–96	+65	0.1			500
			[64]		+ + +	21–51	+2,000 −30,000	1.4			
			[55]		+ + +	31–53			7.3	1 s	1,000
				[65]	+ + +	−3–76	1				0

All the reported techniques, as well as the conditions reported (heating method, level of integration, range of temperature, spatial distribution and power needed), are summarized in a table at the beginning of the paper. Generally, it seems that there is currently no consensus on any given technique that would satisfy all the requirements specified by the complete range of applications; however each of the techniques described here has successfully demonstrated the integration of temperature control for specific applications.

Note also that temperature mapping techniques are beyond the scope of the current paper, for this we refer the reader to the recent review by Gosse, Bergaud and Löw [71]. 

## 2. External Heating

This section describes techniques based on commercial heaters, either to heat liquids prior to being injected into the microsystem, (pre-heated liquids), or the incorporation of commercial components such as Peltier elements. Depending on the final application it is possible to generate either uniform temperature control or temperature gradients, as outlined in the next subsections.

### 2.1. Homogeneous Temperature

**Figure 1:**
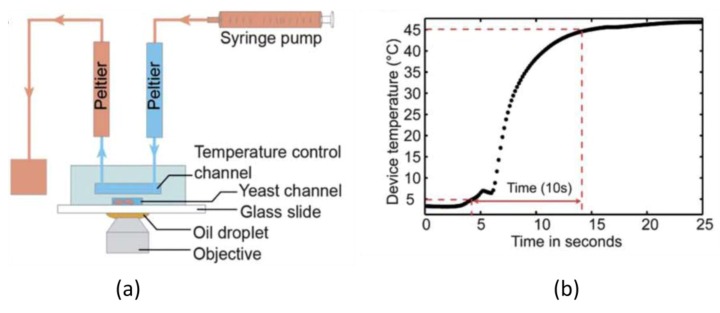
(**a**) A schematic representation of the control device: temperature is set by an external Peltier element; the yeast channel is placed below the temperature control channel (**b**) Temperature *versus* time plot showing a heating rate of 4 °C/s. Reprinted from [37], Copyright 2010, with permission from Elsevier.

A number of techniques using pre-heated liquids have been reported for microfluidic devices. These methods utilize microheaters such as Peltier elements to establish either a uniform temperature or a constant gradient in a given region. Velve Casquillas and co-workers [36,37] developed a disposable polydimethylsiloxane (PDMS) based microfluidic device consisting of two Peltier stages controlling the temperature of the liquid flowing through a control channel (Figure 1(a)). The virtue of PDMS is its relatively low thermal conductivity (0.15 W/mK typically), which allows efficient heat transfer from the source towards the liquid (minimizing energy losses) [43]. This integrated system is capable of reversibly switching between 5 °C and 45 °C in less than 10 s (Figure 1(b)). Changing the direction of the liquid flow through either a cold or hot Peltier using a syringe pump changes the temperature of cells located underneath the temperature control channel. To characterize the temperature response of the chamber, a thin platinum resistance (50 nm) was bonded to the microchannel block. As the electrical resistance of platinum changes nearly linearly with temperature, the authors could record the temperature inside the cell channels by measuring the resistance in the wire.

The previous example shows the potential to exploit external Peltier elements, typically by positioning these elements underneath a microchip. Maltezos and co-workers [2,3] report the use of a microfluidic thermal heat exchanger to cool a Peltier junction and demonstrate rapid heating and cooling of small volumes of solution (typically 0.4 µL). The microfluidic device is able to perform very fast cycling over a temperature range from 22 to 95 °C. The introduction of four parallel Peltier junctions resulted in ramp rates of about 100 °C/s for heating, and 90 °C/s for cooling. In a nutshell, this simple technique represents a miniaturized PCR-on-a-chip system to amplify DNA fragments.

**Figure 2:**
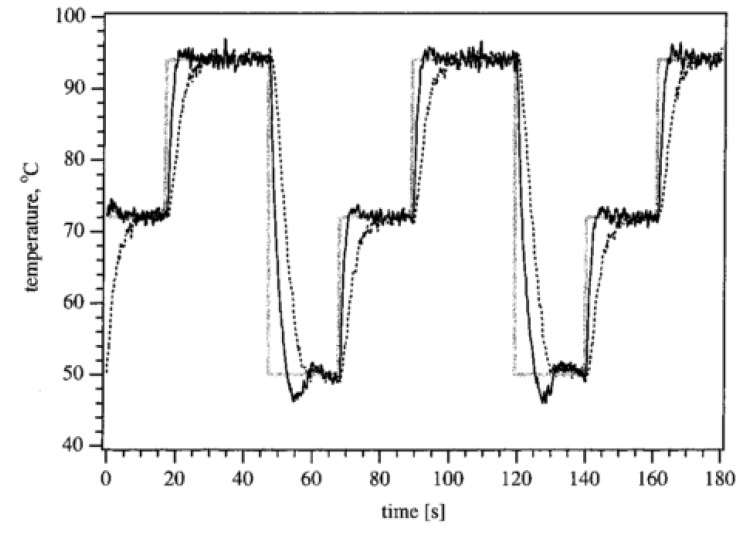
Temperature *versus* time for Polymerase Chain Reaction (PCR) amplification. The gray solid line represents the set-point temperature, the black solid line is the temperature of the bottom Peltier element and the dashed line is the reaction mix temperature. Reprinted with permission from [4]. Copyright 2000 American Chemical Society.

More sophisticated set-ups have been described by Khandurina *et al.* [4] who have developed a device consisting of a compact thermal cycling assembly based on Peltier elements surrounding a microchip gel electrophoresis platform for rapid PCR based analysis. The temperature ramp rates achieved are typically 20-30 °C/s. For amplification, the temperature steps are 94 °C, 50 °C and 72 °C with hold times of 30, 20, 25 s, resulting in ~1.25 min/cycle (Figure 2).

**Figure 3:**
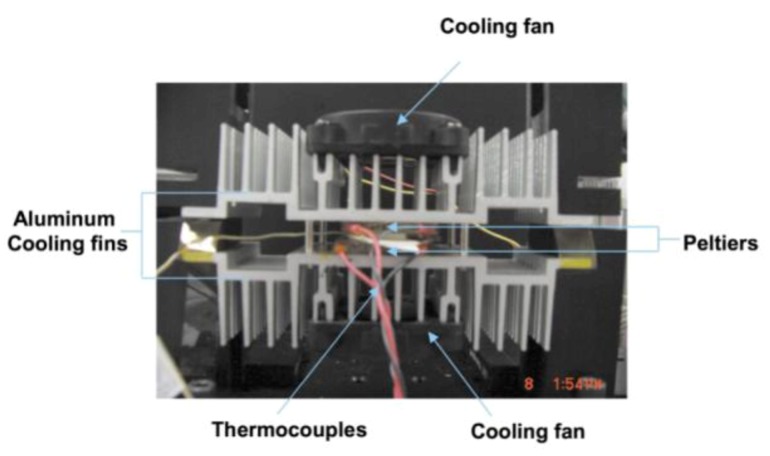
A picture of the chip included in the heating-cooling device. Reprinted with permission from [5]. Copyright 2002, Royal Society of Chemistry.

Along similar lines, Yang *et al.* [5] used a serpentine shaped thin (0.75 mm) polycarbonate PCR micro reactor and demonstrated its detection sensitivity and specificity in amplification of the *E. Coli* K12-specific gene fragment. During thermal cycling, the PCR device is sandwiched between two Peltier elements (Figure 3). The authors performed 30 cycles in 30 min and were able to amplify the K12-specific gene from 10 cells in the presence of 2% blood. Peltier surface and intra-chamber temperatures are transduced by thermocouples which regulate the temperature cycles. Heating rates of 7–8 °C/s and cooling rates of 5–6 °C/s can be achieved using this technique.

Qiu *et al.* [11] described a new method to perform PCR diagnostics based on plastic microfluidic reactors with relatively large volumes (10 to 100 µL). The device is a portable thermal cycler combined with a compact detector for real-time PCR, which can quantify the amount of amplified DNA during an experiment. The chip is located between the master thermoelectric element and a thermal plate. The system achieves a temperature ramp rate of approximately 4 °C/s for heating and 6 °C/s for cooling, and the temperature of the liquid in the reaction chamber follows the set-point temperature with an accuracy of ±0.1 °C up to a temperature of 94 °C.

Maltezos *et al.* [2,3] integrated micro-Peltier junctions of size 0.6 × 0.6 × 1 mm^3^ into their microfluidic device in order to heat and cool nanoliter fluid volumes. These junctions generate a temperature range from −3 °C to 120 °C with an accuracy of about 0.2 °C, and good long-term stability. Temperature rates of 106 °C/s for heating and 89 °C/s for cooling were achieved.

**Figure 4:**
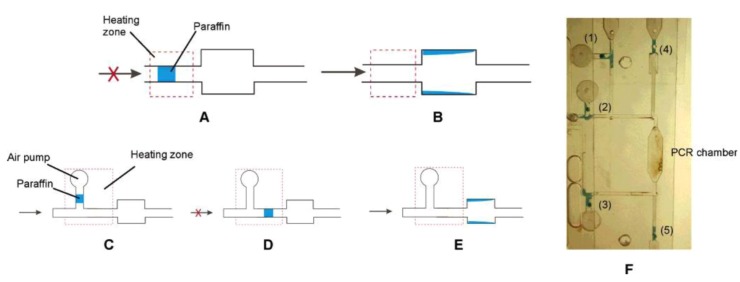
(**a**–**b**) Schematic illustrations of a close-open paraffin microvalve design. (**c**–**e**) An open-close-open microvalve design. (**f**) A photograph of a PCR chamber surrounded by five paraffin-based microvalves: valves 1–3 are open-close valves, and valves 4 and 5 are close-open valves. Reprinted with permission from [72]. Copyright 2004 American Chemical Society.

Apart from PCR applications, Liu *et al.* [72] developed a valving mechanism using paraffin, which undergoes solid-liquid phase transition in response to changes in temperature. As shown in Figure 4, a block of paraffin initially blocks the channel. The paraffin is melted by a heater located directly underneath the chip, and moved downstream by pressure coming from an upstream channel. Once the molten paraffin moves out of the heating zone, it begins to solidify on the wall of a wider channel. The opening of the valve is single use and facilitates transportability in a sealed system. However, the response time of devices mentioned above are of the order of 5–10 s, which is relatively high compared to other systems which bring into play pressure controlled on-off valves [73,74].

Mahjoob *et al.* [7] introduced porous inserts with high temperature conductivity to improve heat transfer by providing a large surface area for a given volume. The system is assembled in three layers: the porous medium is located above an impermeable conductive plate and the microchip is placed underneath this plate. An optimized technique is established based on the effects of several parameters (heat exchanger geometry, conductive plate, porous matrix material used. *etc*.) on the temperature distribution and the power required to circulate the fluid in the heat exchanger. The heating/cooling ramp of the PCR heat exchanger is equal to 150.82 °C/s, which is considerably higher than results reported elsewhere in the literature.

### 2.2. Temperature Gradient

**Figure 5:**
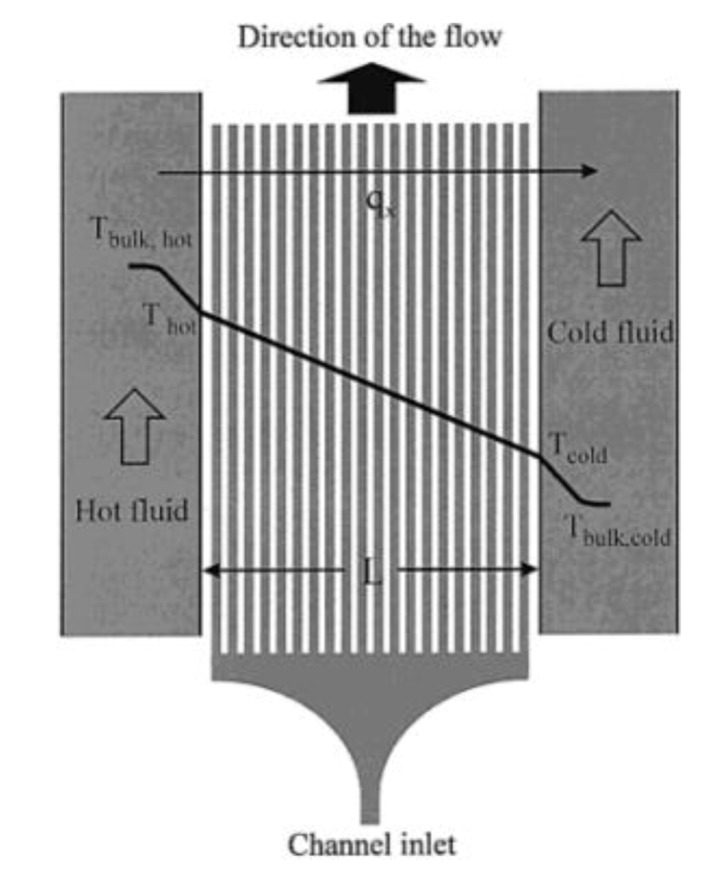
A schematic of the device producing a linear temperature gradient. q_x_ is a representation of the heat flux going from the hot source on the left to the cold one on the right. Reprinted with permission from [38]. Copyright 2002 American Chemical Society.

It is also possible to generate temperature gradients using the pre-heated liquids approach as reported by Mao *et al.* [38]. A linear temperature gradient is generated across dozens of parallel microfluidic channels simultaneously, located in between a hot source and a cold sink separated by a straight wall (Figure 5). The device was manufactured using soft lithographic techniques [39] and its dimensions range from 20 × 7 µm² up to 250 × 7 µm². The linear temperature profile of 5.8 °C/mm depicted in Figure 5 was measured in a microfluidic device composed of eight parallel channels located in between the heating and cooling tubes. A thermocouple is placed at different locations giving rise to the plot presented on Figure 5.

In a similar approach, Matsui and co-workers [16] integrated two Peltier elements to generate a temperature gradient, which can achieve temperature gradients of 13.75 °C/mm across a 4 mm gap. The dimensions of the Peltier elements are 20 mm wide, 40 mm in length and 3.4 mm in height. The authors combine a temperature gradient, an applied electric field and a buffer with a temperature-dependent ionic strength in order to focus analytes by balancing their electrophoretic velocities against the bulk velocity of the buffer containing the analytes (TGF). In 45 s, Oregon Green 488 carboxylic acid is concentrated approximately 30 fold by applying a moderate electrical field of 70 V/cm and a temperature gradient of 13.75 °C/mm across a 4 mm gap.

**Figure 6:**
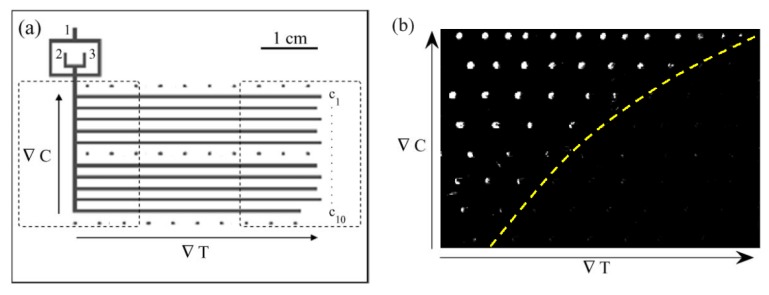
(**a**) Design of the microfluidic device (channel width 500microns). Silicone oil is injected in inlet 1 and aqueous solutions in inlets 2 and 3. The two dotted areas indicate the positions of Peltier modules used to apply temperature gradients. The three lines of dots mark the positions of temperature measurements. (**b**) Example of directly reading out of a solubility diagram. The dotted line surrounding droplets containing crystals gives an estimation of the solubility limit. Reprinted with permission from [35]. Copyright 2007, Royal Society of Chemistry.

Finally, the generation of temperature gradients using Peltier elements can be applied to map-out solubility phase diagrams. Laval *et al.* [35] devised a new microfluidic chip that allows the direct and quantitative reading of two-dimensional solubility diagrams (Figure 6). Firstly, droplets containing a solute with a gradual variation of concentration are stocked on the chip. Crystallization is induced in these droplets by rapid cooling, and finally, a temperature gradient is applied to dissolve crystals in droplets at temperatures higher than their solubility temperature. As a result, they directly sample the solubility boundary between droplets with and without crystals, which gives the solubility temperatures at different concentrations (*i.e*., 2D-readable system: abscissa with temperature, and ordinate with concentration). The temperature field of the chip is controlled by two Peltier elements located underneath a silicon wafer which forms a chip support to optimize thermal transfers, and generates regular temperature gradients of about 0.7 °C/mm along the storage channels. This original technique is simple and cheap and could potentially be used in high throughput studies, given the small amount of reagents needed (around 250 μL).

Peltier elements are widely used to create hot/cold zones, and are able to generate a spatial distribution of temperature with impressive accuracy. However, for many techniques, these elements are not considered as an integral part of the microfluidic chip because of their size, which is typically several millimeters. However, methods have been developed to integrate heating or cooling functionalities directly into microfluidic systems. These approaches are presented in the following sections.

## 3. Integrated Heating

**Figure 7:**
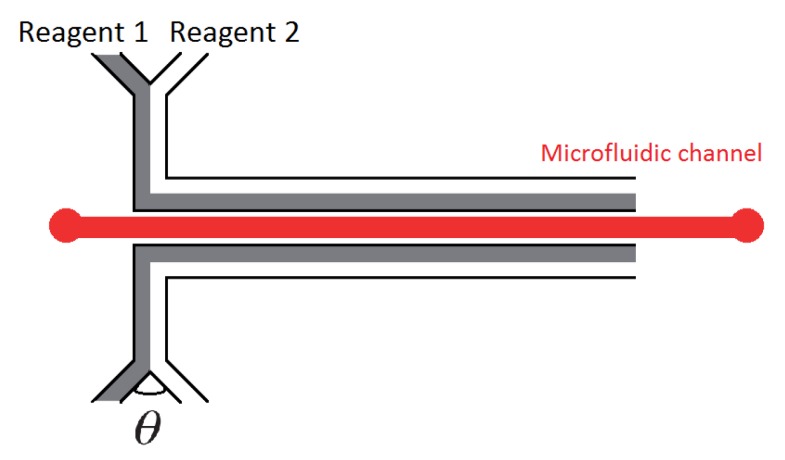
Two reactant channels merging into a temperature control channel, running parallel to the working channel.

We now turn to integrated techniques, from which heat diffuses from/to the integrated heating/cooling source. The first example we present derives from the use of a chemical reaction. In 2002, Guijt *et al.* [65] made use of endothermic and exothermic processes to locally regulate temperature in a microchannel. This method is fully integrated and cost effective with channels of typical dimensions: 54 µm wide and 19 µm deep. For cooling, the evaporation of acetone (Reagent 1) in the air (Reagent 2) is used as an endothermic process. For heating, the dissolution of 97 wt% H2SO4 (Reagent 1) in water (Reagent 2) is used as an exothermic reaction. The central channel (represented in red on Figure 7) is filled with a solution of 1 µM Rhodamine B in water so that the fluorescence gives a direct measurement of the temperature inside the microchannel. Note that heating experiments were conducted in glass-glass channels whereas cooling trials were carried out in PDMS-glass systems. By tuning the flow rate ratio between the two reagents, the authors demonstrate control over the intensity of the reaction and hence the temperature. This approach can achieve temperatures ranging from −3 °C up to 76 °C with ramps about 1 °C/s.

This kind of approach was optimized by Maltezos *et al.* in 2006 [66] for cooling. The authors compared a range of different solvents and angles (θ in the schematic) of the Y-junction, evaporated in a N_2_ flux. They concluded that the most efficient solvent they tested was di-ethyl ether with an angle of 10°, which offers the possibility to cool down to −20°C with a steady state for several minutes. This method is again cheap and clearly suited for microfluidic applications but requires further refinement of the heating control to work efficiently in PDMS channels. 

**Figure 8:**
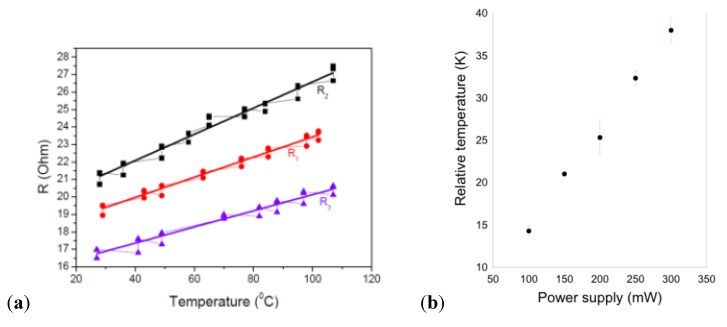
(**a**) Calibration curves: plot of the resistance R *versus* temperature T for the three microheaters. Reprinted from [42], Copyright 2011, with permission from Elsevier. (**b**) Temperature increase as a function of power supply. Reprinted from [45], with kind permission from Springer Science+Business Media.

The following section concerns the most widely reported technique in the literature, based on Joule heating temperature control approaches [20,21,28,29,30,41,42,43,44,45,46]. The technique relies on a simple physical property of conducting metals or liquids. Whichever technique is used to embed heating resistors in a microfluidic system, a linear relationship can be demonstrated between the dissipated power (given by the applied potential and the resistance of the heater) and the heat flux. A stationary temperature profile (Figure 8) can be achieved either by the addition of a heat sink, or by feedback control requiring the integration of a sensor (this point is critical for all techniques in which power is applied—increasing the mean temperature—as opposed to imposing a temperature). In addition, due to the small size of the heaters, the required heating power generated is in the range of 1 W by applying only a few Volts.

Thermal actuation of microfluidic valves by generating a heating pulse has recently been reported. Pitchaimani *et al.* [75] used a PDMS based microfluidic chip to control fluid flow in microchannels. The authors took advantage of constrained deformation in PDMS to develop a thermally actuated plastic microfluidic valve. The fluid flow is controlled through the deflection of a thin elastomeric film, actuated by a temperature-sensitive fluid located inside the valve. Heaters are manufactured by depositing a 100 nm thick gold film onto a cleaned plastic film by sputtering. Depending on the heater power used, the local channel temperature was 10 to 19 °C above the room temperature, enabling control of flow rates from 0.33 to 4.7 µL/min in a 110 µm wide and 45 µm deep microchannel.

**Figure 9:**
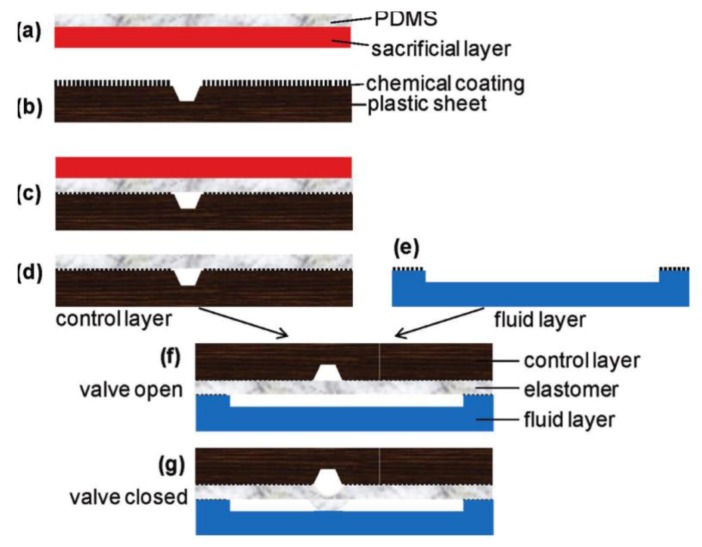
Process ﬂow of bonding a thermoplastic substrate with a polydimethylsiloxane (PDMS) layer (**a**–**d**), followed by additional steps for valve fabrication (**e**–**g**). Reprinted with permission from [76]. Copyright 2011 American Chemical Society.

Similarly, Gu *et al.* [76] used a PDMS based three-layer structure to control the opening/closing of a microchannel (Figure 9). This technique is also applicable to polymethylmethacrylate (PMMA). The valve-containing device can withstand about 700 kPa without delamination, and the PDMS/PMMA bonding strength reaches a plateau when the temperature is higher than 70 °C.

Finally, different temperature profiles may be required: either homogeneous as in PCR applications, or gradient-like for TGF or droplet actuation techniques. In both cases, it may be crucial to perform a temperature profile with the best achievable accuracy, although some applications do not require a sharp control. In order to meet such stringent requirements, different heating techniques and geometries of heaters have been investigated: the use of ionic liquids, *in situ* fabrication of wires and surface patterning of metal resistors using classical microelectronic techniques. These techniques are summarized in two larger categories: the generation of a homogeneous temperature profile and generation of a temperature gradient.

The next two subsections are dedicated to spatial control of the temperature.

### 3.1. Homogeneous Temperature

**Figure 10:**
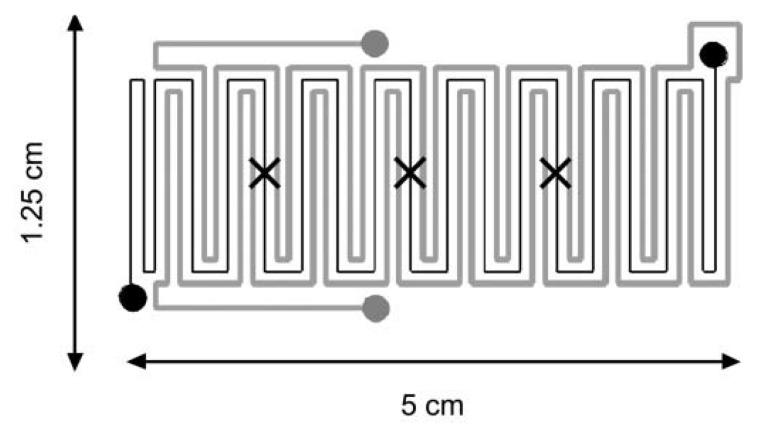
Sketch of the device composed of a working channel (depicted in black) together with parallel channels containing the conductive liquid (depicted in gray). Crosses stand for the position of thermocouples. Reprinted with permission from [41]. Copyright 2004, Royal Society of Chemistry.

To our knowledge, the only reported work using a conductive liquid is from De Mello *et al.* [41]. The authors present a microfluidic device incorporating working channels (sample) with a serpentine-like geometry and parallel channels (Figure 10) in which ionic liquids are Joule heated with an ac current (up to 3.75 kV, f = 50 Hz and P = 1 W). Consequently, the internal temperature can be easily and directly controlled. Temperature measurements were performed using three thermocouples. The ionic liquids used in this experiment were [BMIM][PF6] and [BMIM][Tf2N]. Devices can be heated rapidly or slowly, depending on the applied voltage, and temperatures ranging from 50 °C to 90 °C can be set to within ±0.2 °C.

**Figure 11:**
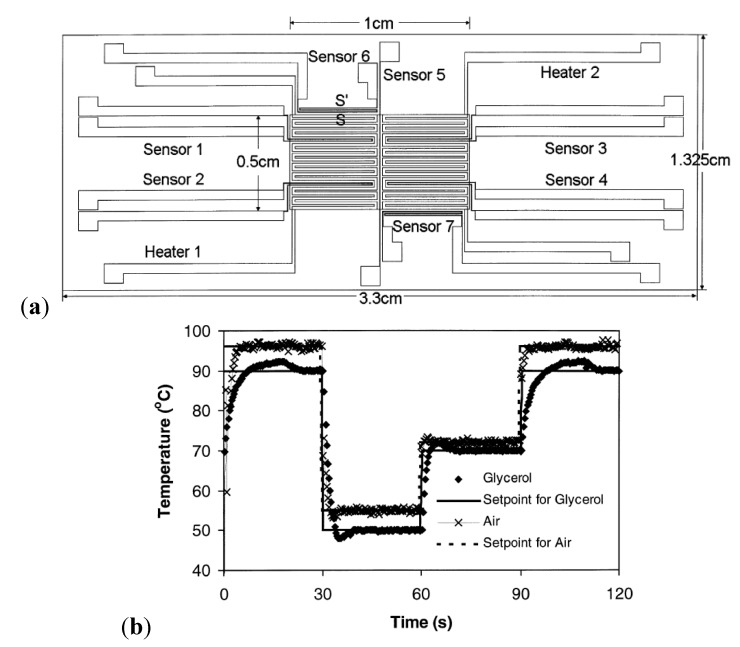
(**a**) Integration of platinum heaters (serpentine-like geometry) together with the integration of sensors. (**b**) Temperature response of the reaction chamber for different fluids, showing the gain scheduling control algorithm efficiency. Reprinted from [44], Copyright 2000, with permission from Elsevier.

The serpentine-like geometry was also studied by Lao *et al.* [44] with integrated platinum heaters and sensors (Figure 11(a)), thermally isolated and digitally feedback controlled allowing a temperature control of ±1 °C and rapid heating/cooling processes: (heating rate of 20 °C/s and cooling rate of 10 °C/s, response time of approximately 5 s). A feedback control, based on a gain scheduling control algorithm, is used to have an improved temperature response inside the chamber. The maximum power required to maintain a 20 µL glycerol solution at 90 °C is 2.2 W. Figure 11(b) shows a good agreement between the chamber temperature and the set point over one cycle, demonstrating a good control of the overshoot.

Based on the same heater geometry, Mavraki *et al.* [42] developed a simple microfluidic chip made of Pyralux™ with a double-sided Cu-clad polyimide (PI) 136 µm thick substrate. PCR, with a fast DNA amplification rate, is performed. The DNA sample flows through the different thermal zones required to perform PCR (denaturation at 95 °C, annealing at 60 °C and extension at 72 °C, see Section 3.2) in a 150 µm wide and 30 µm deep microchannel, completing 25 thermal cycles and resulting in a 2^25^ multiplication factor of DNA. Each thermal zone is about 25 mm × 10 mm. This study shows a characterization of the microheaters used through the resistance *versus* temperature plot (Figure 8(a)). 

Temperature control can be performed using platinum thin layers as heaters and as temperature sensors. Dinca *et al.* [8] presented a micro PCR reactor device using this type of heater. For the fastest experiment, 32 cycles were successfully carried out in less than 25 min, with temperature ramps of 7.7 °C/s for heating and 6.2 °C/s for cooling. Lien *et al.* [9] presented an integrated microfluidic system capable of performing RT-PCR (Reverse Transcription of RNA to DNA previously to PCR: 70 °C during 10 min, 48 °C during 1 h and 95 °C during 15 min) processes for multiple simultaneous detections of four major types of aquaculture disease markers. Bloc platinum resistors are chosen as the material for the micro heaters and the temperature sensors, and gold (Au) metallization is used for the electrical connectors of both the micro temperature sensors and the array-type micro heaters (heating rate 20 °C/s and cooling rate 10 °C/s). 

**Figure 12:**
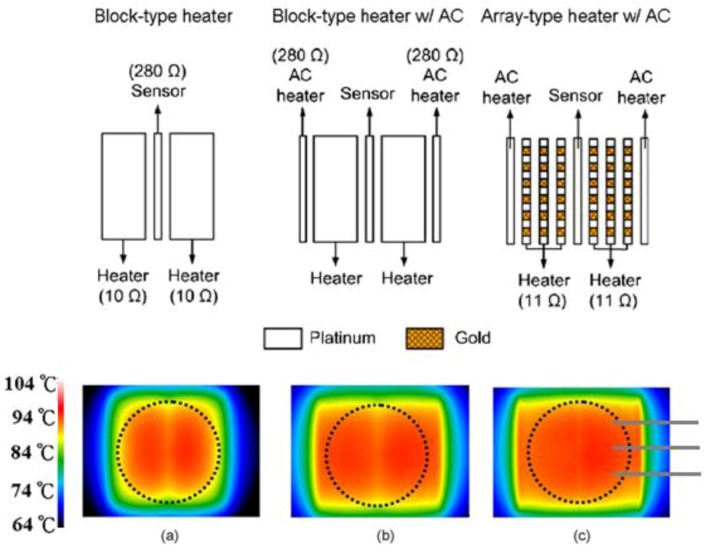
Infrared images of each microthermal cycler without heat sinks at the denaturing temperature. (**a**) 2-D temperature proﬁle of the block-type microheaters. (**b**) 2-D temperature proﬁle of the block-type microheaters with additional side heaters by applying an AC field. (**c**) 2-D temperature proﬁle of the array-type microheaters with AC units. The dotted line shows the location of the PCR reaction chamber. The dimensions of each block in (a) are 2,900 µm × 6,000 µm, which are divided into grids (100 µm × 100 µm) with a spacing of 100 µm in (c). Reprinted from [12], Copyright 2008, with permission from Elsevier.

Hsieh *et al.* [12] performed a rational approach by comparing the temperature response for different geometries of microheaters (Figure 12): two-blocks, two-blocks with additional side heaters, and an array with additional side heaters. Experiments show a temperature homogeneity improvement while increasing the number of heating sources for a given spatial region. An interesting matter raised by the authors is the level of accuracy while stating that the temperature is homogeneous on a whole cavity. As shown in Figure 12(c), it is obvious that a sensor placed at different locations (represented by gray lines) returns an average temperature smoothing the fluctuations along the sensor. Hence these experiments underline that stating a homogeneous temperature requires temperature mapping over the whole region of interest. 

The authors went deeper into their study by investigating other geometries such as serpentine-shape and self-compensated array-type heaters [13]. The aim of the study was to improve the temperature uniformity for PCR applications. Indeed, a homogeneous heater pattern cannot lead to a homogeneous temperature due to side effects, where thermal losses are higher than in the central zone of interest. The authors use electron-beam evaporation and standard lift-off processes to pattern thin-film heaters (90 nm Pt/15 nm Ti), a temperature sensor (90 nm Pt/15 nm Ti) and electrical leads (180 nm Au/20 nm Ti). Results show that a regular array gives a better homogeneity than two-blocks or serpentine, however this can be improved by a self-compensation: the heaters placed at the edges are smaller in order to counter-balance the side effects. The authors tested different self-compensations configurations. The self-compensated heaters happen to give the best uniformity on a selective region, with percentages of the uniform area of 90.3, 99.9 and 96.8 % at 94, 55 and 72 °C respectively, within thermal variation of 1 °C.

**Figure 13:**
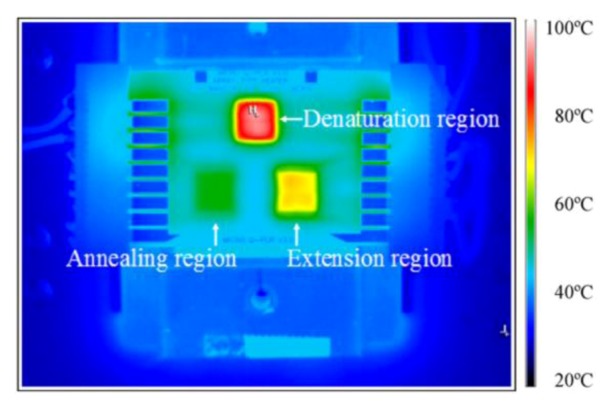
IR images of the device showing the three different temperature zones (5, 72 and 94 °C). Reprinted from [15], Copyright 2004, with permission from Elsevier.

This approach has been valued for PCR amplification by flowing reagents from one region, with a set temperature of 55 °C, to a warmer one (set temperature: 75 °C). The microfluidic system contains three heating regions of different temperatures together with microfluidic channels. The temperature cycling is achieved by making a loop on the three regions. In 2009, Wang *et al.* [15] designed a microchip based on this principle. As shown in Figure 13, they designed three reaction open chambers (5 mm diameter) connected with microfluidic channels. Underneath, three array-type microheaters (Figure 12(c)) are patterned and delivered a homogeneous temperature profile. The liquid is displaced thanks to peristaltic valves [73] in approximately 2 s. A cycle is performed in 110 s. The main advantage of this method is the ease of temperature calibration and thus its precision.

**Figure 14:**
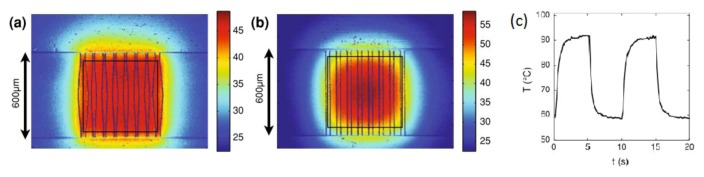
(**a**) Experimental temperature distribution resulting from the optimized resistor, providing a mean temperature in the cavity of 49 °C; (**b**) Experimental temperature distribution in the non-optimized case (*i.e*., with constant-width elements), for a mean temperature into the cavity of 51 °C. (**c**) Experimental mean temperature *versus* time for cycles with a 10 s period and an acquisition frequency of 25 Hz. The transient state lasts approximately 2 s. Reprinted from [45], with kind permission from Springer Science+Business Media.

In the same spirit of shape optimization, Selva *et al.* [29,45] provided shape optimization of heating resistors in order to generate different temperature profiles. Shape optimization was carried out on the heating resistor shape, coupling two numerical tools: a genetic algorithm (NSGAII) [77,78], and a finite element study of the thermal response of the heaters. The resistors are made of chromium 15 nm thick. The typical heating power required is of the order of hundreds of mW. A 600 µm × 600 µm square region is heated at 49 °C with a transient regime of 2.2 ± 0.1 s to reach an asymptotic state (90% of the asymptotic value is reached in about 1 s, which is much faster than a Peltier heater), see Figure 14(a,b) for which it is clear that side effects have to be compensated by thinner resistors at the edges. The cycling temperature was demonstrated as having good stability over time, provided a heat sink is placed below the cavity (Figure 14(c)). 

By patterning the substrate with an optimized resistor, it is possible to generate a homogeneous temperature within a cavity with great accuracy and with short response times (standard deviation below 1 °C and asymptotic regime reached after 2.2 s).

**Figure 15:**
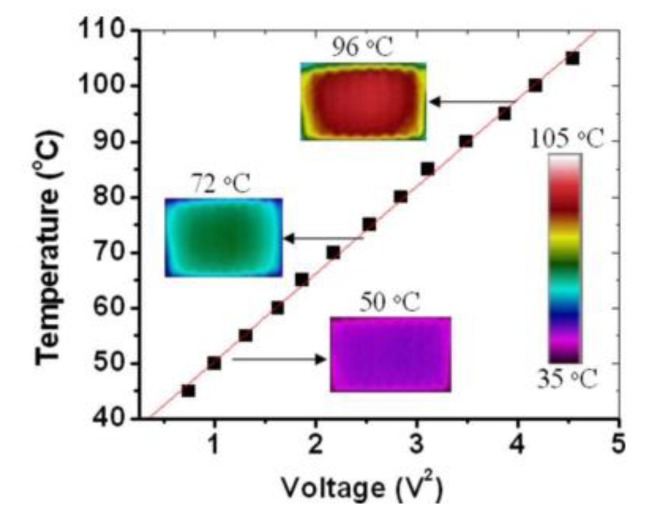
Temperature in the working channel as a function of the squared input voltage. The three insets are IR images illustrating the spatial distribution. Reprinted with permission from [67]. Copyright 2009, American Institute of Physics.

The last reported technique is the integration of metal wires. Wu *et al.* [67] designed a microheater and also thermal sensor directly by injecting silver paint (or other conductive materials) into a PDMS microchannel. In this study, they use SPI silver paste diluted by SPI thinner (ratio 1:3) followed by an ultrasonic bath treatment. The paste is injected in the channel and then heated to vaporize the solvent in three steps: 60 °C, 100 °C and 150 °C. The calibration curve Resistance *vs.* Temperature is done with an IR camera and reveals a good spatial homogeneity in the middle of the serpentine. It also shows a good linearity in the 45–105 °C range. They achieved a heating rate of 20 °C/s and a steady state error of about ±0.5 °C. With an applied voltage varying from 0.9 to 2.2 V, the authors obtained a temperature from 45 to 110 °C (Figure 15). Moreover, by measuring the resistance of a thinner wire, they could deduce its temperature. Finally, by designing a double serpentine (a large one for heating and a thin one for sensing), they created a microheater and a thermal sensor. Adding air-cooling channels, LabView voltage and air pressure controls (with a PID module), they finally designed a 25 × 25 mm² temperature controller that can be bonded under a micro-chip. One of the advantages of this technique is the low cost of the device. 

### 3.2. Temperature Gradient

For given applications (e.g., droplet actuation, Soret effect, TGF, *etc*.) it is necessary to generate temperature gradients, either in a controlled way (controlled shape of the temperature profile) or not. 

In the field of droplet-based microfluidics, a first application is focused on the displacement of a droplet in a capillary (1D geometry). Nguyen *et al.* [22] presented both theoretical and experimental results of thermocapillary effects of a liquid plug in a long capillary, subject to a transient temperature gradient generated by a resistive heater. The transient temperature gradient spreads in the capillary wall much slower than the droplet itself. Consequently, the plug moves out of the high-gradient region and decelerates. Jiao *et al.* [23] reported the reciprocating thermocapillary motion of a liquid plug located in a capillary and positioned between two heaters. The model shows the coupling effect between the surface tension driven movement of the plug and the heat transfer in the capillary wall. The temperature gradients, generated by the two heaters, cause a liquid motion. Finally, Shen *et al.* [14] investigated the physical mechanisms affecting migration of droplets due to thermocapillarity. A constant thermal gradient (up to 4.21 °C/mm) is generated by powering a metal heater stripe at one edge of the chamber, and cooling at the opposite edge by circulating coolant through a brass heat sink. The results of this study shed light on the critical role of mechanical or chemical hysteresis, and highlight the need to minimize power requirements in microfluidic devices.

**Figure 16:**
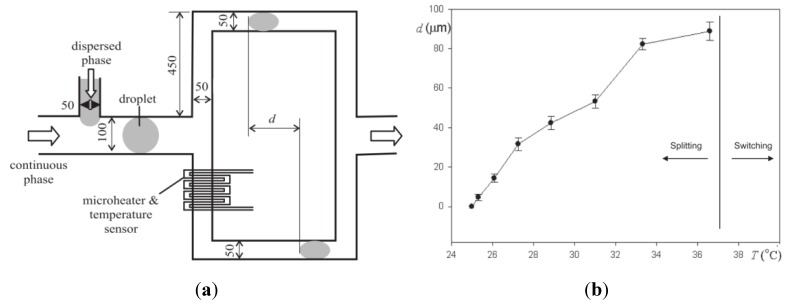
(**a**) A schematic view of the microfluidic device (dimensions in µm). (**b**) Variation of the delay distance d with temperature. Reprinted with permission from [18]. Copyright 2099, Institute of Physics.

Another 1D droplet handling can be performed using the integration of a serpentine-like micro-heater which locally generates a temperature gradient together with a local decrease in the continuous phase viscosity. Considering such an integration in a 1D geometry, it is possible to control the breakup or switching of a droplet arriving in a T-junction as reported by Yap *et al.* [18,19]. The authors present a thermal control technique for microdroplets at a bifurcation, using an integrated microheater which induces simultaneously thermocapillarity and a reduction in fluidic resistance in one of the branches (Figure 16(a)). Droplet breakup and switching are demonstrated within a temperature range of 25–38 °C (Figure 16(b)), which enables dealing with biological samples.

**Figure 17:**
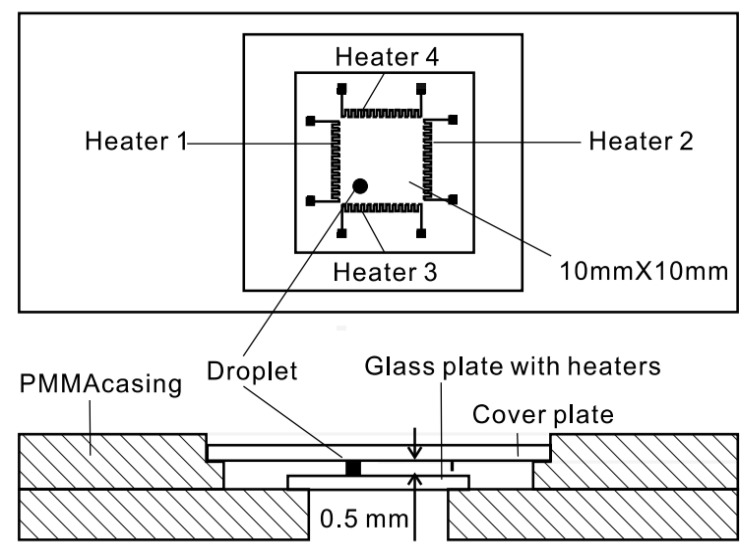
Top and lateral views of the device showing four heaters placed along a square, generating temperature gradients, for which both shape and magnitude influence the heating power of each single heater. Reprinted with permission from [21]. Copyright 2008, Institute of Physics.

Jiao *et al.* [20,21] presented a device with four integrated heaters providing temperature gradients for droplet-based microfluidic systems (Figure 17). The heaters are structured on a glass wafer of a 10 mm × 10 mm square region and are made of thin-film titanium and platinum. The maximum heating power of each heater is equal to 0.5 W.

**Figure 18:**
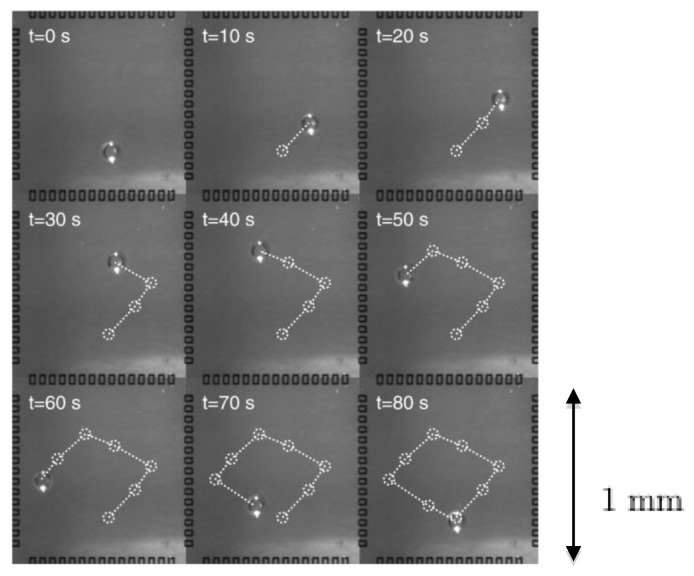
Succession of droplet positions by spatially varying the temperature gradient in time, (duration 80 s). Reprinted with permission from [21]. Copyright 2008, Institute of Physics.

In such a configuration, it is possible to drive droplets by imposing a succession of different temperature gradients along the 2D substrate. The four microheaters actuated independently generate variable surface tension gradients. The droplet can be positioned anywhere in the channel depending on the strength of individual heaters (Figure 18).

**Figure 19:**
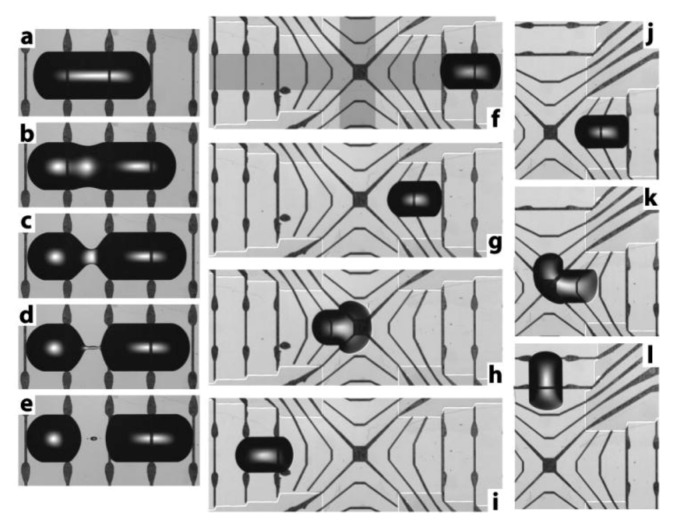
(**a**–**e**) Thermally induced splitting of a dodecane drop on a partially wetting stripe (w = 1,000 µm). The voltage applied to the microheater (155 Ω) was 2.5 V. The images were recorded at t = 0, 6.0, 7.5, 8.0, and 8.5 s. (**f**–**i**) Dodecane drop propelled through an intersection outlined by the dark gray pattern (w = 1,000 µm, time lapse 104 s). (**j**–**l**) Dodecane drop turning a 90° corner (time lapse 164 s). Reprinted with permission from [24]. Copyright 2003, American Institute of Physics.

At a more integrated level, Darhuber *et al.* [24,25,26,27] developed a system with thin Ti metallic microheaters (thickness 100 nm, length 3 mm and 0.8 mm width, and 500 nm SiO2 layer deposited for electrical heaters isolation ) coupled to a chemical patterned glass substrate and electronic actuation. The typical range of applied power for a single microheater is 5–200 W (maximum output voltage 10 V; maximum output current 90 mA). Based on thermocapillary actuation, they controlled, with a great accuracy, the formation, 2D displacement, coalescence and breakup of droplet on demand [27] (Figure 19). The initial volume of liquid is 3–16 µL.

**Figure 20:**
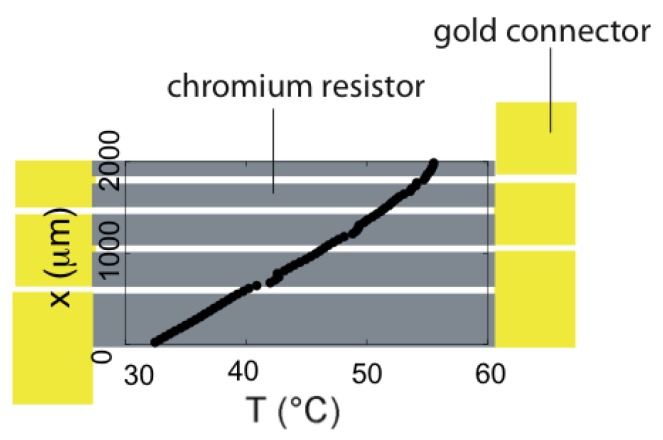
Top view of an output resistor geometry obtained performing shape optimization, made of chromium resistors (in gray) and gold connectors (in yellow). The experimental temperature profile along the cavity shows a linear dependence of the temperature with the x-axis.

Selva *et al.* [29] also reported shape optimization on resistors (chromium 15 nm thick, connected by gold wires 150 nm thick) to generate a linear temperature profile, as sketched in Figure 20. Applying a power ranging from 200 to 500 mW, an intense temperature gradient (up to 11 °C/mm with a standard deviation of approximately 1%) is generated (Figure 20). The transient regime of application of the gradient lasts about 250 ms.

**Figure 21:**
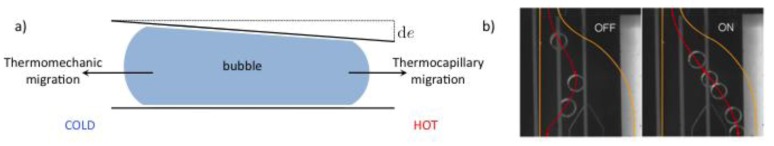
(**a**) Sketch of the competition between thermocapillary and thermomechanical effects on bubble migration. Reprinted with permission from [28]; Copyright 2011, American Institute of Physics. (**b**) Images of a 300 µm diameter bubbles inside a switching device: (**left**) without actuation, and (**right**) with a 4 °C/mm temperature gradient. Reprinted with permission from [30]. Copyright 2010, Royal Society of Chemistry.

Using this resistor pattern, another phenomenon has been emphasized by Selva *et al.* [28]: thermomechanical effects due to PDMS dilation with increasing temperature. The authors present studies of pancake-like shaped bubbles in a Hele-Shaw cell, submitted to a temperature gradient [29]. Under such a confinement, there are mainly two competing mechanisms arising from the temperature gradient: thermocapillarity, and the thermal dilation of the PDMS cavity (Figure 21(a)). A theoretical model predicts the cavity dilation to be the dominant effect, which happens to be in excellent agreement with experimental results, inducing a bubble motion toward the cold region of the cavity. According to this study, Selva *et al.* [30] report a method for bubble and droplet displacement, switching (Figure 21(b)) and trapping based on a thermomechanical effect. This technique presents a high level of integration with low applied voltage (~10 V) and low power consumption (<0.4 W). This work clearly highlights for the first time competing phenomena involved in microfluidics when changing the temperature.

**Figure 22:**
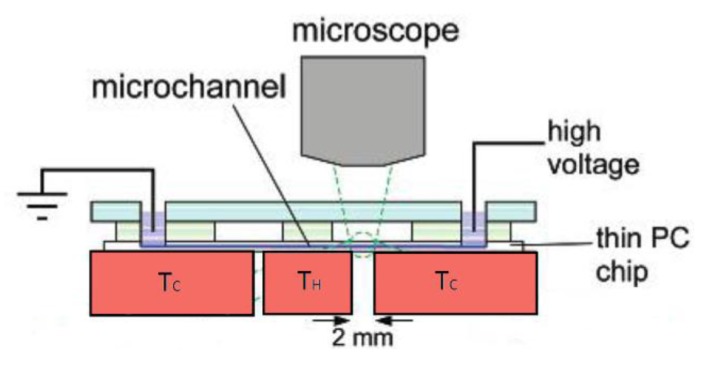
Schematic drawing of the Temperature Gradient Focusing apparatus. Reprinted with permission from [46]. Copyright 2002 American Chemical Society.

In order to generate a temperature gradient, copper blocks can also be integrated within a microsystem. Ross *et al.* [17] described such a system in which a temperature gradient is generated for TGF purposes (see Section 5). The device consists of two copper blocks set to different temperatures in order to generate a temperature gradient across a 2 mm gap microfluidic channel (Figure 22). The system is based on TGF, where temperature gradients of 25 °C/mm are produced by thermally anchoring a thin polycarbonate microchannel chip to alternately heated or cooled copper blocks. The technique is demonstrated for a large variety of analytes (fluorescent dyes, amino acids, DNA, proteins, *etc*.) and is capable of more than 10000-fold concentration of a dilute analyte.

**Figure 23:**
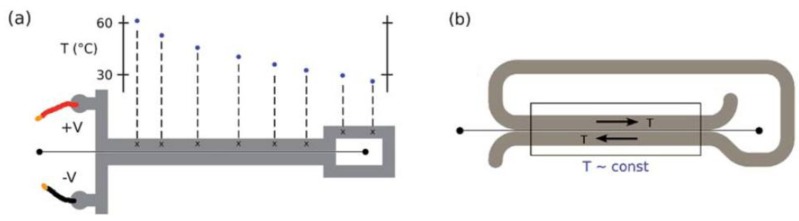
(**a**) Plot of the temperature along the channel surrounded by channels filled with an epoxy. (**b**) A schematic view of a device that is able to create a constant temperature. Reprinted with permission from [43]. Copyright 2010, Royal Society of Chemistry.

An interesting technique of embedded heaters is reported by Vigolo *et al.* [43]. The authors used a silver-filled epoxy (Epo-Tek_H20S, Epoxy Technology) that can be injected and solidified in a microfluidic chip, in parallel channels geometry. Applying an input current, both sides of a microchannel were heated by Joule effect. Depending on the geometry of the channels, either the control of a temperature gradient (Figure 23(a)) or the maintenance of a constant temperature (Figure 23(b)) can be achieved. This approach presents a fully embedded technique to control temperature, and permits working continuously from 25 °C to 75 °C in a PDMS based microfluidic (accuracy ±2–3 °C). In the transient regime, the temperature increases within 10–20 s and reaches a stable value in less than one minute. A thermocouple in contact with a thin glass cover slip was used to measure the temperature. Authors could finally obtain the temperature of the strip by taking into account the thermal conductivity, thickness and cross-sectional area of the glass slide. 

This technique can be combined with the pre-heated liquid technique as reported by Vigolo *et al.* [79] for thermophoresis studies (see Section 5). The authors describe a method for selective driving of particles towards either the hot or the cold side by adding specific electrolytes to their initial solution. The authors used a microfluidic device where temperature gradients were established by combining pre-heated liquid or epoxy resistors on either sides of the microchannel. Experiments bring into play the use of polystyrene beads of 477 nm in diameter in the presence of 100 mM NaCl with a flow rate of 0.01 µL/min, and show the accumulation of particles on the cold side by fluorescence measurements.

**Figure 24:**
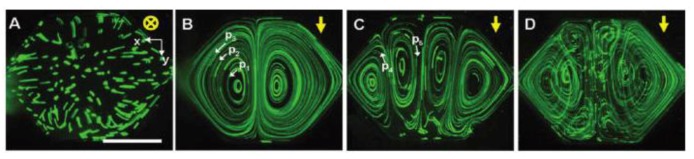
Natural convection-driven ﬂows in a chamber. (**a**–**d**) Flow trajectories of ﬂuorescent microparticles taken for 35 s. Measured maximum temperatures, Tmax, in (a) to (d) are 52, 51, 46, and 50 °C, respectively. The yellow arrows indicate the gravity direction, and the white arrows depict the ﬂow direction of the individual ﬂuorescent particles of 8 µm diameter. Scale bar, 1 mm. Reprinted with permission from [33]. Copyright 2009 American Chemical Society.

Temperature gradient can also be used to generate natural convection for mixing purposes. Rapid and homogeneous mixing is difficult to achieve in microscale. Indeed, even if diffusion processes are favored in miniature fluidic systems, a pure diffusion-based mixing can be very inefficient, especially in solutions where macromolecules have a diffusion coefficient several orders of magnitude lower than that of most liquids. However, micromixing in chambers remains challenging even though many in-line micromixers have been developed and successfully demonstrated [32,34]. Kim *et al.* [33] presented an effective technique that enables micromixing in a microfluidic chamber without using a pump. By using natural convection in conjunction with alternating heating of two heaters (Figure 24), efficient micromixing is achieved. Heaters are made in a Ti/Pt alloy formed by a lift-off process, whose dimensions are typically 20 nm/100 nm in thickness. Fluorescent microbeads of 8-µm diameter were used as flow tracers to measure the flow speed at steady state. Standard deviation 
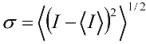
 was used to determine the degree of mixing in the chamber, where I is the normalized intensity of each pixel.

## 4. Electromagnetic Radiation

Up to now, we have seen that temperature control can be performed either in an external way or by integrating heating resistors. In both cases, heat diffuses from a source towards the liquid of interest. This section is devoted to techniques able to heat the liquid in the bulk, *i.e.*, without using a thermal source. 

### 4.1. Microwaves

Microwave dielectric heating is a fundamentally different approach because of its preferential heating capability and non-contact delivery energy. Induced and intrinsic dipole moments align themselves with a time-varying electric field (from 3 to 20 GHz). The energy associated is viscously dissipated as heat into the surrounding media with no interference from the substrate materials. Compared to conventional techniques, enhanced thermocycling rates and reduced reaction times can be achieved [54]. Heating can also be made spatially selective by confining the electromagnetic fields to specific regions thanks to integrating miniaturized microwave heating elements. The use of microwave heating has been demonstrated for a variety of applications including drug discovery [62], PCR [63] isolation of DNA and heating of biological cells [60].

**Figure 25:**
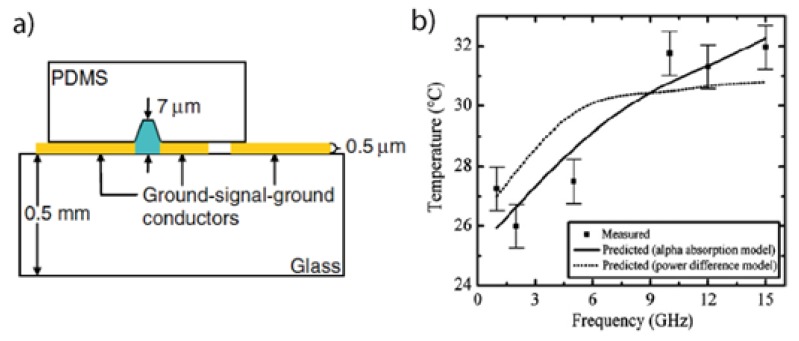
(**a**) Lateral view of a coplanar waveguide transmission line (yellow) surrounding a PDMS microchannel (blue). (**b**) Measured temperature (squares) of an aqueous solution as a function of frequency compared with two theoretical models (solid and dashed lines). Reprinted with permission from [59]. Copyright 2007, Institute of Physics.

Microwave power at several GHz is delivered to the channel by transmission line integrated in the microfluidics device. In most cases, a coplanar waveguide configuration is used. Shah *et al.* [59] carried out a study where a microchannel fabricated in PDMS [39] was aligned with a thin film microwave transmission line in a coplanar waveguide (CPW) configuration. A schematic of the device is shown in Figure 25(a). The CPW was comprised of a 140 μm wide signal conductor separated by a 25 μm gap on either side from 300 μm wide ground conductors. The CPW conductors, 1.5 cm long, were formed by thermally evaporating Cr/Au (10 nm/500 nm) on a 0.5 mm thick glass wafer and using a standard lift-off metallization process.

The device is characterized by the scattering (S) parameter and the temperature. The S-parameters are the transmission and reflection coefficients. The fluid temperature is obtained by measuring the temperature dependent fluorescence intensity of a dilute fluorophore, Rhodamine B, added to the fluid [46]. Figure 25(b) shows temperature *vs.* various microwave frequencies. Experimental data are compared to a theoretical model based on classical microwave absorption theory. They observed a 0.88 °C·mW^−1^ temperature rise at 12 GHz and 0.95 °C·mW^−1^ temperature rise at 15 GHz. In agreement with the theory, they concluded that the temperature rise of the fluid is predominantly due to the absorbed microwave power. These results have been confirmed by recent works done on microwave dielectric heating of fluids [56].

**Figure 26:**
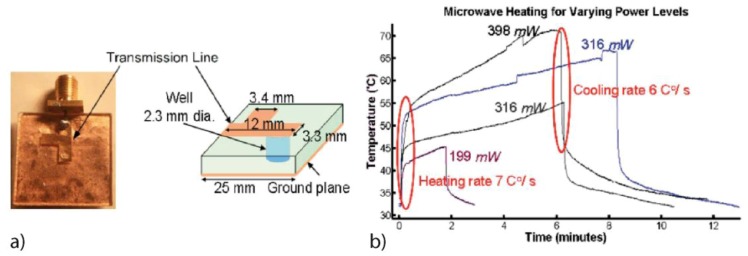
(**a**) Device schematic. (**b**) Temperature *versus* time for varying power levels. Reprinted with permission from [56]. Copyright 2009, American Institute of Physics.

Kempitaya *et al.* [56] generated microwaves with millimetric copper strip transmission line (copper thickness 130 µm) put on top of a 1 mm thick polycarbonate substrate with a 2.3 mm diameter drilled well. Figure 26(a) shows such a device; the volume test chamber corresponds to 4.1 µL. They investigated the performance of microwave heating on a high temperature range, up to 70 °C. The thermal response is recorded as a function of the applied microwave power for a prolonged period of time. 

They report two distinct heating/cooling behaviors (Figure 26(b)). During the first 30 s when the power is turned on (off), the heating (cooling) rate is very fast around 7 °C/s (6 °C/s). The temperature is controlled by the heating/cooling of the microfluidic well (small thermal mass). However after this initial step, the heating/cooling rate decreases significantly, less than 0.1 °C/s. The substrate of the device, with a larger thermal mass, controls the temperature rise. A finite element transient heat transfer modeling matches the experimental results and gives the temperature profile along the well. Because of heat loss through the copper electrode the temperature of the liquid is only uniform over more than 50% of the chamber. 

In the same spirit Shaw *et al.* [57] improved the integrated microwave heating device by adding air impingement cooling in order to do rapid PCR amplification. Indeed a previous study performed in 2004 showed the capability of doing PCR in a 15 mL reaction volume but the cooling was not integrated and required manual transfer into a thermally controlled block for each annealing step [58].

**Figure 27:**
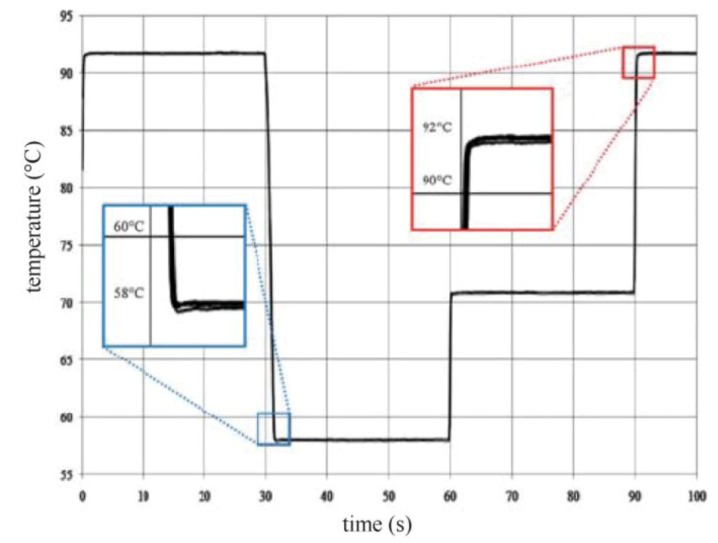
Thermal cycling profile based on selected temperatures of 92, 58 and 71 °C. Reprinted with permission from [57]. Copyright 2010, Royal Society of Chemistry.

Using feedback controlled microwave heating coupled with air impingement cooling, the system showed minimal thermal overshoot or undershoot at any of the three set temperatures (see Figure 27). Once the microwave system had reached a set temperature the variation was less than ±0.1 °C. The ramp rates for heating and cooling exceeded 65 °C/s, allowing very fast transitions between temperatures. In conclusion, this study enabled 28 cycles to be performed in 42 min. This is one order of magnitude faster than current commercial systems.

Microwaves heaters can be used to set the temperature for a PCR reaction. Orlling *et al.* [58] designed a millifluidic tool to prove this concept for samples volumes from 2.5 to 15 mL. At this scale, Joule heating processes loose in efficiency due to a small surface to volume ratio. Consequently, the authors take advantage of the *in situ* heating of the device thanks to microwaves. Cycles in only 200 s are achievable for such a large amount of liquid thanks to extremely fast and smooth heating and cooling. Downscaling was performed by Fermer *et al. *[63] on volumes of about 100 µL, allowing a cycle duration of 144 s with a power of 100 W. Recently, Shaw *et al.* [57] illustrated the viability of this technique at the microfluidic scale. A glass system is composed of a reaction chamber of 0.7 µL, which is supplied by a microchannel to bring the DNA sample and the PCR mix. They performed 90 s cycles with heating and cooling rates as fast as 65 °C/s.

**Figure 28:**
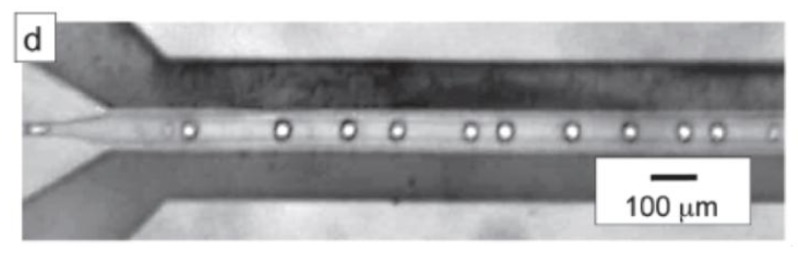
Picture of the device where the dark regions are the metal lines and the gray region in the middle is the channel. Reprinted with permission from [64]. Copyright 2009, Royal Society of Chemistry.

In the context of droplet-based microfluidic, Issadore *et al.* [64] reported the first experiment on microwave heating made on droplets. They used a microfluidic device that integrates a flow-focusing drop maker; drop splitters (Figure 28), and metal electrodes to locally deliver microwave power.

The devices are fabricated using PDMS on-glass drop-based microfluidics. The metal electrodes are directly integrated into the PDMS device using a low-melt solder fill technique [61]. The microwaves are generated with a voltage-controlled oscillator and amplified with a power amplifier. Copper wires approximately 2 mm in length connect the sub miniature assembly connector to the metal electrodes in the PDMS device. The electronics operate at 3.0 GHz. The temperature change of the drops is measured by observing the temperature-dependent fluorescence of cadmium selenide nanocrystals dispersed in the drops. With such a device they demonstrated characteristic heating times as short as 15 ms to steady-state temperature changes as large as 30 °C above the base temperature of the microfluidic device. In terms of applications the authors claim that setting the base temperature of the oil to 65 °C, they could cycle the temperature from 65 °C to 95 °C as required for PCR.

**Figure 29:**
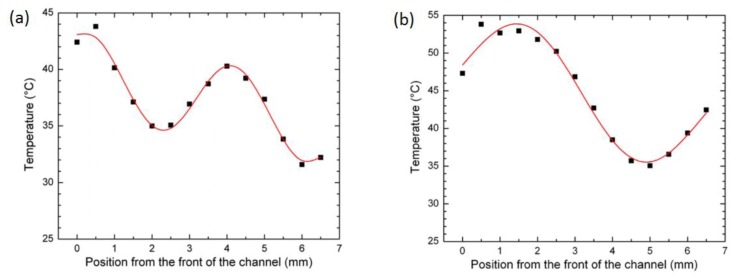
Measured temperature *versus* distance along the microchannel of an aqueous solution for two microwave excitation frequencies: (**a**) 19 GHz and (**b**) 12GHz. The solid red line is a theoretical temperature ﬁt to the data points in squares. Reprinted with permission from [55]. Copyright 2010, Institute of Physics.

Up to now microwave heating has been used to generate homogeneous temperature and a fast heating rate. However spatial and temporal temperature gradients are also of primary interest in the field of enzymatic activity, thermodynamics, kinetics of molecular association, TGF and droplet handling to cite but a few. In their study, Shah *et al.* [55] used large interference effects to make a linear temperature gradient. Interference effects are produced by superposition of a sinusoidal and two exponential temperature distributions. Temperature extremes of 31 °C and 53 °C at the minimum and maximum of the sinusoid were established within 1 s. The sinusoid also produced a quasi-linear temperature gradient along a 2 mm distance with a slope of 7.3 °C·mm^−1^ (see Figure 29).

### 4.2. Use of a Laser

With the development of microelectronics, the laser diode became a cheap and reliable technology. The laser is now an easy and simple way to optically interact with a material. It is thus a rational approach in order to heat precisely low volumes. The spatial precision is easily around a micrometer and the volumes employed in microfluidics allow low consumption of products. Even if it is not an integrated method (for the moment, the laser remains out of the chip), it offers a cheap and spatially reconfigurable heat source that can be precisely addressed.

**Figure 30:**
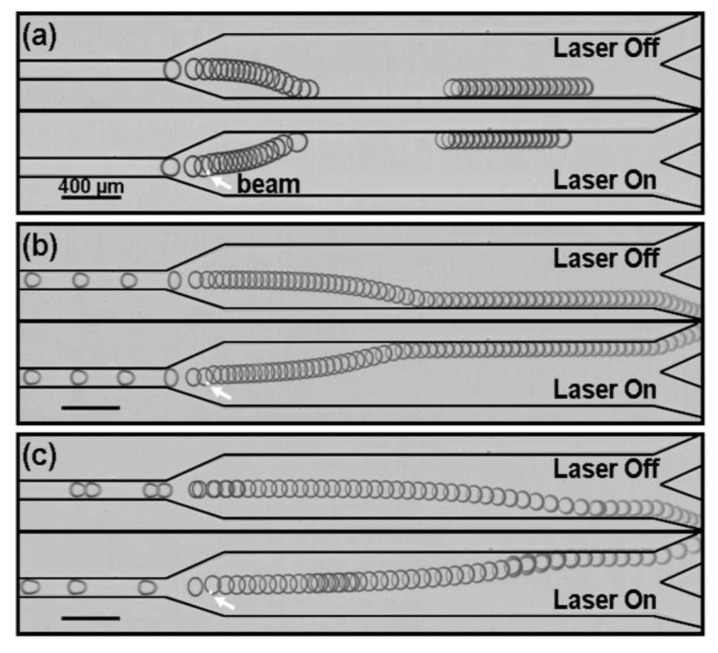
Superposition of successive frames illustrating drop switching by local thermocapillary actuation. The arrow indicates the laser location observed by the ﬂuorescence of the water-dye solution. (**a**) Qo/Qw = 5/0.05, U = 2.6 mm/s, R = 60 µm, P = 58 mW, frame rate 50 frames/s. (**b**) Qo/Qw = 10/0.1, U = 6.1 mm/s, R = 54 µm. P = 111 mW, frame rate 100 frames/s. (**c**) Qo/Qw = 15/0.2,U = 13.0mm/s, R = 55 µm. P = 159 mW, frame rate 100 frames/s. Flow rates are expressed in µl/min. Reprinted with permission from [68]. Copyright 2008, American Institute of Physics.

In their study, Robert de Saint Vincent *et al.* [68] used a laser as a heating source. The experiment consisted of focusing a continuous Argon-ion laser (wavelength in vacuum λ_0_ = 514.5 nm) on a PDMS microchannel by using an inverted microscope. The aqueous phase is heated by adding 0.1 wt% of fluorescein (which gives an optical absorption of the solution α = 1.18 cm^−1^, thus absorbing the laser radiation). The maximum thermal gradient that can be obtained at the edge of the beam is given by P/w_0_ (P is the power of the laser) and reaches (for P around 100 mW) 10 to 20 °C/mm. The temperature gradient localized at the front of a droplet creates a surface tension gradient and induces droplet displacement in order to sort them. In Figure 30, one can see that for three different speeds (a–c), a 100% sorting of droplets is accomplished (water + dye drops in oil). When the laser is switched on, it locally heats the liquids (on an area about the beam waist) and thus creates a surface tension difference and induces a flow that moves the drop due to Marangoni stress. Baroud *et al.* [80] used the same concept with a flow focusing geometry to control the production of droplets.

Kim *et al.* [69] also used a laser in order to heat nanoliter drops and to perform real-time PCR. Their laser is an infrared diode at a wavelength of 1.46 μm, which corresponds to the first overtone of the O-H stretch vibration of liquid water. The beam size is about 200 μm with a 10× objective in order to heat 300 μm diameter droplets. To perform the PCR, they use two powers: 25 and 50 mW to achieve 60 and 95 °C. The accuracy of the steady state is less than 1 °C. They perform a complete amplification in 20 s. This method can be coupled to a powerful laser with a microlens array in order to realize real-time PCR over hundreds of droplets.

**Figure 31:**
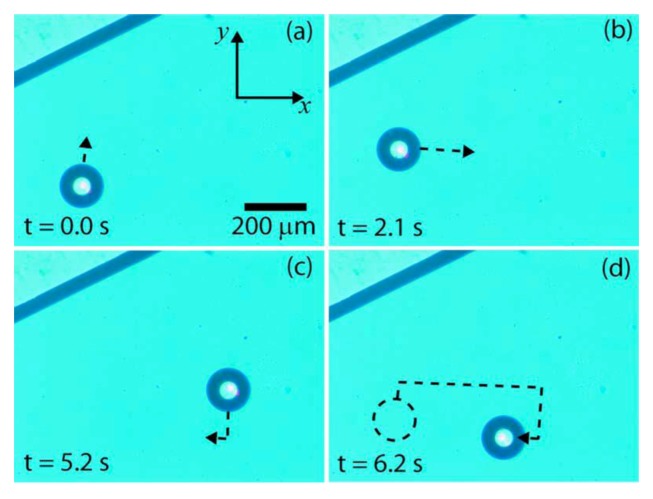
Optically actuated thermocapillary movement of an air bubble in silicone oil. A 114-µm-diameter (1.0 nL) bubble is trapped in the thermal trap created by a laser focused on the absorbing substrate (**a**). The oil/air meniscus can be seen at the top left. The bubble follows the position of the laser spot, as it is scanned in the positive y direction (**b**) and then in the positive × direction (**c**). The bubble is then moved in the negative y and negative x directions (**d**). A dashed circle indicates the initial bubble position, and a dashed line indicates the approximate trajectory of the bubble. Reprinted with permission from [70]. Copyright 2007, American Institute of Physics.

Ohta *et al.* [70] used this tool to control the displacement of a gas bubble in silicon oil *via* thermocapillary forces. Contrary to other works, the temperature gradient is generated by the laser absorption in the silicon substrate and not in the liquid. In order to create optically actuated thermocapillary forces, they used an absorbing substrate consisting of a 0.85-mm-thick glass slide coated with a 100-nm-thick layer of indium tin oxide, followed by a 1-μm-thick layer of hydrogenated amorphous silicon (*a*-Si:H), which absorbs light in the visible and UV wavelengths. The laser power used is 10 mW, and its wavelength is 635 nm. The magnification is 20 and the beam obtained is a 6 μm-diameter spot on the surface of the absorbing substrate. They managed gradients in the surrounding oil up to 4 °C/mm. As one can see in Figure 31, the bubble is first trapped at the hottest point (because the bubble moves toward the warmest region) and then the laser spot is moved. This technique shows a good control of the displacement and moreover, it allows the use of fluids without any optical restrictions.

Another application stemming from the use of a laser was reported by Weinert *et al.* [81]. The authors were able to pump selected parts of a fluid film along the path of a moving warm spot which was generated by an infrared laser focus. The maximal temperature increase in the local spot is about 10 °C in water, corresponding to pump speeds of 150 µm/s. The enhanced temperature in the spot leads to a reduced viscosity: experiments confirm that the fluid motion results from the dynamic thermal expansion in a gradient of viscosity. Consequently, the fluid moves opposite to the spot direction. Using this technique, the authors were able to pump nanoparticles over millimeters through a gel, and mixing was demonstrated for fluids sandwiched between untreated and unstructured, disposable microscope cover slips.

Hettiarachchi *et al. *[82] presented an optical microfluidic platform for performing real-time polymerase chain reactions of breast cancer cell DNA within droplet-in-oil microreactors. Droplet manipulation and rapid thermal cycling were achieved by using a low power (20–40 mW) infrared laser (lambda_0 = 1,460 nm). Droplet temperatures were calibrated based on fluorescence measurements of SYBR Green as well as amplification efficiency. The typical droplet diameter is about 200 µm and fluorescence images are acquired at a 400 ms acquisition time. The illumination source was blocked with an automated shutter to avoid photobleaching. The authors also addressed the problematic of achieving spatial homogeneity. Indeed, the authors initially ran into problems with temperature non-uniformity across the droplets that impacts amplification efficiency. As a result, a 4-f lens configuration was used to defocus the laser beam to achieve a more uniform temperature distribution. 

## 5. Applications

This section introduces a series of applications to illustrate the efficiency of reported heating techniques. We have tried as much as is possible to cover a range of different fields from the chemical, physical and biological sciences, without being exhaustive in any one domain; our purpose is rather to explore the diversity of microfluidic applications exploiting thermal control. The strategies and approaches vary from one application to another; while spatial temperature homogeneity is imperative for many biological applications; a rough temperature gradient is sufficient for most applications in digital microfluidics and a net temperature gradient is required for Temperature Gradient Focusing (TGF) and thermophoresis.

For biological applications, two main requirements have been reported: (1) maintaining the temperature at 37 °C to keep cells alive [83], the effect of temperature on the response of cells to stimuli can be obviously studied, however a homogeneous temperature is required; or (2) controlled temperature cycling for Polymerase Chain Reactions. In both cases, spatial homogeneity of the temperature profile is desirable. Polymerase Chain Reaction (PCR) is widely used in molecular biology to amplify target DNA *in vitro *[1]. A PCR cycle usually consists of three discrete steps, each step being highly temperature sensitive. In the case of DNA, a denaturation step performed at 94–96 °C causes DNA melting yielding single-stranded DNA molecules. Subsequently, an annealing step is carried out at 50–65°C: during which the polymerase attaches to a single-strand and begins DNA formation. Finally, the elongation or extension step is performed at 72 °C and DNA polymerase synthesizes a new DNA strand complementary to the single-strand template. Since PCR steps are short, the duration of a total amplification process with a commercially available thermal cycler is limited by the heating/cooling rates. Consequently, a thermal technique is needed to rapidly increase the heating/cooling cycling speed. The number of groups working on PCR worldwide is significant given the important social impact of this technique for example in the field of medical diagnostics. This paper does not aim to review all the systems that have been developed but rather to present specific approaches that have been reported [30,79]. Two key strategies have been adopted to facilitate thermal cycling: the PCR process is either performed in a single chamber where temperature is modulated over time (single heater device), or three independent heating zones in the microsystem (multiple heater devices) are integrated. In the latter case, solutions carrying the reagents either flow through the three regions with residence times matching the corresponding PCR step (multiple heater device), or are incubated in a preset temperature chamber for the required time. 

Another approach is the generation of temperature gradients to drive droplets. Indeed, the use of droplet-based microfluidics [31] is largely presented as a viable alternative to handle small volumes (typically a few hundreds of a nanoliter). Micro droplets can be considered as perfect discrete biological reactors as they minimize cross-contamination, provided the partition coefficients prevent diffusion through the droplet interface [84]. Droplets also exhibit mixing by rolling recirculation that homogenizes the content, and can thus be considered as *bona fide* microreactors. The growing demand for handling picoliter to nanoliter volumes of biological samples has driven the development of droplet techniques where a variety of processes, including mixing, splitting and heating are efficiently controlled: these approaches have been designated as digital microfluidics. Such a prospective, demands tight control of fundamental operations such as droplet merging, fission, transport, exchanges, redirection, and storage. This has rekindled interest in the Marangoni surface effect, which refers to tangential stresses induced along an interface by a surface tension gradient [85]. The primary mechanism at play here is thermocapillarity, and bubbles or droplets are driven by the flow resulting from a gradient along their surface. Methods to induce temperature gradients in droplet systems are described and relatively coarse temperature gradients may be sufficient to drive droplets in these microsystems. This approach is an alternative to the Electrowetting On Dielectric (EWOD) technique [86] which refers to Maxwell stress applied to the triple line contact. The local electric field changes the local curvature and induces droplet motion. Although in the thermocapillary and EWOD, droplet motion is achieved with potentials of about 10 V, EWOD requires optimizing dielectric thicknesses and properties [87].

Conversely, the following applications, which focus on chemical applications, demand a net temperature gradient, *i.e.*, a constant temperature gradient. The detection of chemical species at small concentrations (nanomolar or lower) in small volumes (typically a few microliters or less) is a core functionality of miniaturized bioanalytical devices. Temperature Gradient Focusing (TGF), on the other hand, involves the application of a temperature gradient across a microchannel or capillary. With an appropriate buffer, the temperature differential creates a gradient in both the electric field and the electrophoretic velocity. Ionic species can thus either be concentrated by balancing the electrophoretic velocity against the bulk flow, or separated according to their individual electrophoretic mobilities. In other words, tuning the temperature allows modulation of the electrophoretic mobility at will. Another application centered on the migration of small particles, is thermophoresis. Thermophoresis (Soret effect) is the phenomenon wherein small particles suspended in a fluid with a temperature gradient, experience a force in the direction opposite to that gradient [88,89]. This effect can be used to drive particles in microfluidic devices and presents several advantages in terms of selectivity, due to its sensitivity to particle interfacial properties. 

Finally, thermal control has found application in a broad gamut of diverse fields such as the development of efficient and rapid mixing techniques (for example solutions with low Reynolds number), or the screening of solubility diagrams to study protein crystallization.

## 6. Conclusions

Applications concerned with the control of temperature are numerous and focus on physical, chemical and biotechnological issues. This article shows the great variety of technologies that have been developed to achieve integrated temperature control. All these techniques present different advantages or drawbacks in terms of easiness of integration, cost, area of control, accuracy of the control, *etc*. that are summarized in the following table. This table underlines the fact that despite there being no paradigm to implement microheaters, a huge improvement has been performed in terms of level of integration, cost and response time showing very good ability to integrate the focused application. However, an important feature has been emphasized by several authors [12,13,28,29,30,45,82] concerning temperature measurement. Indeed, it has been shown that to generate a homogeneous temperature profile within a cavity or within a droplet, whole mapping of the temperature has to be performed since a sensor simply outputs a mean value. Even if a small standard error is not crippling for PCR applications, it can be so when using cells where biological activity is extremely sensitive to slight temperature variations. The development of new heating techniques will require focus on generating such accuracy.

As stated in the introduction, several strategies have been adopted to control the temperature within a microsystem, and most of the focused applications have been successfully achieved. It is thus difficult to isolate a technique that could be a paradigm. However, for some applications it is possible to extract a technique—that seems to be more accurate and show a high level of integration. In the case of droplet handling, Darhuber *et al.* [24,25,26,27], using chemical patterning and thin metal wires, were able to dispense, merge, break and drive droplets on a substrate. In this case a temperature pulse generates a temperature gradient sufficient to perform the expected functions; *i.e.*, an accurate temperature profile is not necessary. For biological applications requiring a homogeneous temperature at the cavity level, Joule heating connected with shape optimization to avoid side effects has been shown to be very efficient with a high level of integration [12,13,45]. At the droplet level, IR heating is also very efficient, provided the beam is defocused to guarantee spatial temperature homogeneity in the droplet [82]. Finally, for screening solubility diagrams for which a temperature gradient is required, apart from the use of Peltier, techniques have been developed at a high level of integration such as shape optimization [29], such an approach could be used in the future. All these technologies are mature; nevertheless one may envisage in the future a technology embedding a heating source such as a conductive PDMS. Cong and Pan [90] succeeded in performing a thermal conductivity of about 80 W·m^−1^·K^−1^ by adding 21% of silver powder. Such technology would avoid extra steps in the microfabrication process but would require optimization of thicknesses in order to proceed to optical measurements.

## References

[1] Bartlett J.M.S., Stirling D. (2003). A short history of the polymerase chain reaction. Meth. Mol. Biol..

[2] Maltezos G., Gomez A., Zhong J., Gomez F.A., Scherer A. (2008). Microfluidic polymerase chain reaction. Appl. Phys. Lett..

[3] Maltezos G., Johnston M., Taganov K., Srichantaratsamee C., Gorman J. (2010). Exploring the limits of ultrafast polymerase chain reaction using liquid for thermal heat exchange: A proof of principle. Appl. Phys. Lett..

[4] Khandurina J., McKnight T.E., Jacobson S.C., Waters L.C., Foote R.S., Ramsey J.M. (2000). Integrated system for rapid PCR-based DNA analysis in microfluidic devices. Anal. Chem..

[5] Yang J., Liu Y., Rauch C.B., Stevens R.L., Liu R.H., Lenigk R., Grodzinski P. (2002). High sensitivity PCR assay in plastic micro reactors. Lab Chip.

[6] Hua Z., Rouse J.L., Eckhardt A.E., Srinivasan V., Pamula V.K., Schell W.A., Benton J.L., Mitchell T.G., Pollack M.G. (2010). Multiplexed real-time polymerase chain reaction on a digital microfluidic platform. Anal. Chem..

[7] Mahjoob S., Vafai K., Beer N.R. (2008). Rapid microfluidic thermal cycler for polymerase chain reaction nucleid acid amplification. Int. J. Heat Mass Transfer.

[8] Dinca M.P., Gheorghe M., Aherne M., Galvin P. (2009). Fast and accurate temperature control of a PCR microsystem with a disposable reactor. J. Micromech. Microeng..

[9] Lien K.Y., Lee S.-H., Tsai T.-J., Chen T.-Y., Lee G.-B. (2009). A microfluidic-based system using reverse transcription polymerase chain reactions for rapid detection of aquaculture diseases. Microfluid. Nanofluid..

[10] Wang W., Li Z., Yang Y., Guo Z. Droplet Based Micro Oscillating Flow-Through PCR Chip. Proceedings of the 17th IEEE International Conference on Micro Electro Mechanical Systems (MEMS).

[11] Qiu X., Mauk M.G., Chen D., Liu C., Bau H.H. (2010). A large volume, portable, real-time PCR reactor. Lab Chip.

[12] Hsieh T.-M., Luo C.-H., Huang F.-C., Wang J.-H., Chien L.-J., Lee G.-B. (2008). Enhancement of thermal uniformity for a microthermal cycler and its application for polymerase chain reaction. Sens. Actuator. B.

[13] Hsieh T.-M., Luo C.-H., Wang J.-H., Lin J.-L., Lien K.-Y., Lee G.-B. (2009). Enhancement of thermal uniformity for a microthermal cycler and its application for polymerase chain reaction. Microfluid. Nanofluid..

[14] Shen K., Chen X., Guo M., Cheng J. (2005). A microchip-based PCR device using flexible printed circuit technology. Sens. Actuator. B.

[15] Wang J.-H., Chien L.-J., Hsieh T.-M., Luo C.-H., Chou W.-P., Chen P.-H., Chen P.-J., Lee D.-S., Lee G.-B. (2009). A miniaturized quantitative polymerase chain reaction system for DNA amplification and detection. Sens. Actuator. B.

[16] Matsui T., Franzke J., Manz A., Janasek D. (2007). Temperature gradient focusing in a PDMS/glass hybrid microfluidic chip. Electrophoresis.

[17] Ross D., Locascio L.E. (2002). Microfluidic temperature gradient focusing. Anal. Chem..

[18] Yap Y.F., Tan S.H., Nguyen N.T., Murshed S.M.S., Wong T.N., Yobas L. (2009). Thermally mediated control of liquid microdroplets at a bifurcation. J. Phys. D Appl. Phys..

[19] Ting T.H., Yap Y.F., Nguyen N.-T., Wong T.N., Chai J.C.K. (2006). Thermally mediated breakup of drops in microchannels. Appl. Phys. Lett..

[20] Jiao Z., Huang X., Nguyen N.-T., Abgrall P. (2008). Thermocapillary actuation of droplet in a planar microchannel. Microfluid. Nanofluid..

[21] Jiao Z., Huang X., Nguyen N.-T. (2008). Manipulation of a droplet in a planar channel by periodic thermocapillary actuation. J. Micromech. Microeng..

[22] Nguyen N.T., Huang X.Y. (2005). Thermocapillary effect of a liquid plug in transient temperature fields. Jpn. J. Appl. Phys..

[23] Jiao Z.J., Nguyen N.T., Huang X.Y., Ang Y.Z. (2007). Reciprocating thermocapillary plug motion in an externally heated capillary. Microfluid. Nanofluid..

[24] Darhuber A.A., Valentino J.P., Davis J.M., Troian S.M. (2003). Microfluidic actuation by modulation of surface stresses. Appl. Phys.Lett..

[25] Darhuber A.A., Valentino J.P. (2003). Thermocapillary actuation of droplets on chemically patterned surfaces by programmable microheater arrays. J. Microelectromech. Syst..

[26] Darhuber A.A., Davis J.M., Troian S.M. (2003). Thermocapillary actuation of liquid flow on chemically patterned surfaces. Phys. Fluids.

[27] Darhuber A.A., Valentino J.P., Troian S.M. (2010). Planar digital nanoliter dispensing system based on thermocapillary actuation. Lab Chip.

[28] Selva B., Cantat I., Jullien M.-C. (2011). Temperature-induced migration of a bubble in a soft microgravity. Phys. Fluids.

[29] Selva B., Marchalot J., Jullien M.-C. (2009). An optimized resistor pattern for temperature gradient control in microfluidics. J. Micromech. Microeng..

[30] Selva B., Miralles V., Cantat I., Jullien M.-C. (2010). Thermocapillary actuation by optimized resistor pattern: bubbles and droplets displacing, switching and trapping. Lab Chip.

[31] Guo M.T., Rotem A., Heyman J.A., Weitz D.A. (2012). Droplet microfluidics for high-throughput biological assays. Lab Chip.

[32] Stroock A.D., Dertinger S.K.W., Ajdari A., Mezic I., Stone H.A., Whitesides G.M. (2002). Chaotic mixer for microchannels. Science.

[33] Kim S.-J., Wang F., Burns M.A., Kurabayashi K. (2009). Temperature-programmed natural convection for micromixing and biochemical reaction in a single microfluidic chamber. Anal. Chem..

[34] Liu R.H., Stremler M.A., Sharp K.V., Olsen M.G., Santiago J.G., Adrian R.J., Aref H., Beebe D.J. (2000). Passive mixing in a three-dimensional serpentine microchannel. J. Microelectromech. Syst..

[35] Laval P., Lisai N., Salmon J.-B., Joanicot M. (2007). A microfluidic device based on droplet storage for screening solubility diagrams. Lab Chip.

[36] Velve Casquillas G., Fu C., Le Berre M., Cramer J., Meance S., Plecis A., Baigl D., Greffet J.-J., Chen Y., Piel M., Tran P.T. (2011). Fast microfluidic temperature control for high resolution live cell imaging. Lab Chip.

[37] Velve Casquillas G., Costa J., Carlier-Grynkorn F., Mayeux A. (2010). A fast microfluidic temperature control device for studying microtubule dynamics in fission yeast. Method. Cell Biol..

[38] Mao H., Yang T., Cremer P.S. (2002). A microfluidic device with a linear temperature gradient for parallel and combinatorial measurements. J. Am. Chem. Soc..

[39] Xia Y., Whitesides G.M. (1998). Soft lithography. Angew. Chem. Int. Ed..

[40] Maltezos G., Johnston M., Scherer A. (2005). Thermal management in microfluidics using micro Peltier junctions. Appl. Phys. Lett..

[41] De Mello A.J., Habgood M., Lancaster N.L., Welton T., Wooton R.C.R. (2004). Precise temperature control in microfluidic devices using Joule heating of ionic liquids. Lab Chip.

[42] Mavraki E., Moschou D., Kokkoris G., Vourdas N., Chatzandroulis S., Tserepi A. (2011). A continuous flow µPCR device with integrated microheaters on a flexible polyimide substrate. Procedia Eng..

[43] Vigolo D., Rusconi R., Piazza R., Stone H.A. (2010). A portable device for temperature control along microchannels. Lab Chip.

[44] Lao A.I.K., Lee T.M.H., Hsing I.-M., Ip N.Y. (2000). Precise temperature control of microfluidic chamber for gas and liquid phase reactions. Sens. Actuator. A.

[45] Selva B., Mary P., Jullien M.-C. (2010). Integration of a uniform and rapid heating source into microfluidic systems. Microfluid. Nanofluid..

[46] Ross D., Gaitan M., Locascio L.E. (2001). Temperature measurement in microfluidic systems using a temperature-dependent fluorescent dye. Anal. Chem..

[47] Oda R.P., Strausbauch M.A., Huhmer A.F., Borson N., Jurrens S.R., Craighead J., Wettstein P.J., Eckloff B., Kline B., Landers J.P. (1998). Infrared-mediated thermocycling for ultrafast polymerase chain reaction amplification of DNA. Anal. Chem..

[48] Ferrance J.P., Wu Q., Giordano B., Hernandez C., Kwok Y., Snow K., Thibodeau S., Landers J.P. (2003). Developments toward a complete micro-total analysis system for Duchenne muscular dystrophy diagnosis. Anal. Chim. Acta.

[49] Giordano B., Ferrance J., Swedberg S., Hulhmer A., Landers J. (2001). Polymerase chain reaction in polymeric microchips: DNA amplification in less than 240 seconds. Anal. Biochem..

[50] Ke C., Berney H., Mathewson A., Sheehan M. (2001). Rapid amplification for the detection of Mycobacterium tuberculosis using a non-contact heating method in a silicon microreactor based thermal cycler. Sens. Actuator. B.

[51] Jagannathan H., Yaralioglu G., Ergun A., Khuri-Yakub B. Acoustic Heating and Thermometry in Microfluidic Channels. Proceedings of IEEE the Sixteenth Annual International Conference on Micro Electro Mechanical Systems (MEMS-03 Kyoto).

[52] Kondoh J., Shimizu N., Matsui Y., Sugimoto M., Shiokawa S. (2009). Development of temperature-control system for liquid droplet using surface Acoustic wave devices. Sens. Actuator. A.

[53] Yaralioglu G. (2011). Ultrasonic heating and temperature measurement in microfluidic channels. Sensors Sens. Actuator. A.

[54] Bykov Y.V., Rybakov K.I., Semenov V.E. (2001). High-temperature microwave processing of materials. J. Phys. D Appl. Phys..

[55] Shah J.J., Geist J., Gaitan M.A. (2010). Microwave-induced adjustable nonlinear temperature gradients in microfluidic devices. J. Micromech. Microeng..

[56] Kempitiya A., Borca-Tasciuc D.A., Mohamed H.S., Hella M.M. (2009). Localized microwave heating in microwells for parallel DNA amplification applications. Appl. Phys. Lett..

[57] Shaw K.J., Docker P.T., Yelland J.V., Dyer C.E., Greenman J., Greenway G.M., Haswell S.J. (2010). Rapid PCR amplification using a microfluidic device with integrated microwave heating and air impingement cooling. Lab Chip.

[58] Orrling K., Nilsson P., Gullberg M., Larhed M. (2004). An efficient method to perform milliliter-scale PCR utilizing highly controlled microwave thermocycling. Chem. Commun..

[59] Shah J.J., Sundaresan S.G., Geist J., Reyes D.R., Booth J.C., Rao M.V., Gaitan M. (2007). Microwave dielectric heating of fluids in an integrated microfluidic device. J. Micromech. Microeng..

[60] Geist J.J., Gaitan M. (2007). Microwave power absorption in low-reflectance, complex, lossy transmission lines. J. Res. Natl. Inst. Stand. Technol..

[61] Siegel A.C., Shevkoplyas S.S., Weibel D.B., Bruzewicz D.A., Martinez A.W., Whitesides G.M. (2006). Cofabrication of electromagnets and microfluldic systems in poly(dimethylsiloxane). Angew. Chem. Int. Ed..

[62] Kappe C.O., Dallinger D. (2006). The impact of microwave synthesis on drug discovery. Nat. Rev. Drug Discov..

[63] Fermer C., Nilsson P., Larhed M. (2003). Microwave-assisted high-speed PCR. Eur. J. Pharm. Sci..

[64] Issadore D., Humphry K.J., Brown K.A., Sandberg L., Weitz D.A., Westervelt R.M. (2009). Microwave dielectric heating of drops in microfluidic devices. Lab Chip.

[65] Guijt R.M., Dodge A., van Dedem G.W.K., de Rooij N.F., Verpoorte E. (2003). Chemical and physical processes for integrated temperature control in microfluidic devices. Lab Chip.

[66] Maltezos A., Rajagopal A. (2006). Scherer. Evaporative cooling in microfluidic channels. Appl. Phys. Lett..

[67] Wu J., Cao W., Chen W., Chang D.C., Sheng P. (2009). Polydimethylsiloxane microfluidic chip with integrated microheater and thermal sensor. Biofluidics.

[68] Robert de Saint Vincent M., Wunenburger R., Delville J.-P. (2008). Laser switching and sorting for high speed digital microfluidics. Appl. Phys. Lett..

[69] Kim H., Vishniakou S., Faris G.W. (2009). Petri dish PCR: Laser-heated reactions in nanoliter droplet arrays. Lab Chip.

[70] Ohta A.T., Jamshidi A., Valley J.K., Hsu H.-Y., Wu M.C. (2007). Optically actuated thermocapillary movement of gas bubbles on an absorbing substrate. Appl. Phys. Lett..

[71] Gosse C., Bergaud C., Löw P. (2009). Molecular probes for thermometry in microfluidic devices. Top. Appl. Phys..

[72] Liu R.H., Yang J., Lenigk R., Bonanno J., Grodzinski P. (2004). Self-contained, fully integrated biochip for sample preparation, polymerase chain reaction amplification, and DNA microarray detection. Anal. Chem..

[73] Unger M.A., Chou H.-P., Thorsen T., Scherer A., Quake S.R. (2000). Monolithic microfabricated valves and pumps by multilayer soft lithography. Science.

[74] Kartalov E.P., Scherer A., Quake S.R., Taylor C.R., Anderson W.F. (2007). Experimentally validated quantitative linear model for the device physics of elastomeric microfluidic valves. J. Appl. Phys..

[75] Pitchaimani K., Sapp B.C., Winter A., Gispanski A., Nishida T., Fan Z.H. (2009). Manufacturable plastic microfluidic valves using thermal actuation. Lab Chip.

[76] Gu P., Liu K., Chen H., Nishida T., Fan Z.H. (2011). Chemical-assisted bonding of thermoplastics/elastomer for fabricating microfluidic valves. Anal. Chem..

[77] Deb K. (2001). Multi-Objective Optimization Using Evolutionary Algorithms.

[78] Deb K., Pratap A., Agarwal S., Meyarivan T. (2002). A fast and elitist multiobjective genetic algorithm: NSGA-II. IEEE Trans. Evol. Comput..

[79] Vigolo D., Rusconi R., Stone H.A., Piazza R. (2010). Thermophoresis: Microfluidics characterization and separation. Soft Matter.

[80] Baroud C.N., Delville J.-P., Gallaire F., Wununburger R. (2007). Thermocapillary valve for droplet production and sorting. Phys. Rev. E.

[81] Weinert F.M., Braun D. (2008). Optically driven fluid along arbitrary microscale patterns using thermoviscous expansion. J. Appl. Phys..

[82] Hettiarachchi K., Kim H., Faris G.W. (2012). Optical manipulation and control of real-time PCR in cell encapsulating microdroplets by IR laser. Microfluid. Nanofluid..

[83] Hung P.J., Lee P.J., Sabounchi P., Lin R, Lee L.P. (2005). Continuous perfusion microfluidic cell culture array for high-throughput cell-based assays. Biotechnol. Bioeng..

[84] Mary P., Studer V., Tabeling P. (2008). Microfluidic droplet-based liquid-liquid extraction. Anal. Chem..

[85] Davis S.H. (1987). Thermocapillary instabilities. Annu. Rev. Fluid Mech..

[86] Mugele F., Baret J.-C. (2005). Electrowetting: From basics to applications. J. Phys. Condens. Matter.

[87] Moon H., Cho S.K., Garrell R.L., Kim C.J. (2002). Low voltage electrowetting-on-dielectric. J. Appl. Phys..

[88] Vigolo D., Brambilla G., Piazza R. (2007). Thermophoresis of microemulsion droplets: Size dependence of the Soret effect. Phys. Rev. E.

[89] Piazza R., Guarino A. (2002). Soret effect in interacting micellar solutions. Phys. Rev. Lett..

[90] Cong H., Pan T. (2008). Photopatternable conductive PDMS materials for microfabrication. Adv. Funct. Mater..

